# Convalescent plasma therapy for COVID-19 prophylaxis in adults early post-hematopoietic stem cell transplantation: one-year outcomes from a randomized controlled trial

**DOI:** 10.3389/fimmu.2025.1626775

**Published:** 2025-11-28

**Authors:** Yigeng Cao, Qiang Li, Wenwen Guo, Mingyue Xu, Kuo Fang, Fengjiao Wang, Rongli Zhang, Lin Li, Jialin Wei, Zhenyun Liu, Chen Liang, Weihua Zhai, Qiaoling Ma, Xin Chen, Wenbin Cao, Donglin Yang, Yi He, Aiming Pang, Sizhou Feng, Mingzhe Han, Jiali Sun, Erlie Jiang

**Affiliations:** 1 Institute of Hematology & Blood Diseases Hospital, Chinese Academy of Medical Sciences & Peking Union Medical College, Tianjin, China; 2 State Key Laboratory of Experimental Hematology, National Clinical Research Center for Blood Diseases, Tianjin, China

**Keywords:** convalescent plasma, COVID-19, hematopoietic stem cell transplantation, prophylaxis, randomized controlled trial

## Abstract

To evaluate the efficacy of COVID-19 convalescent plasma (CCP) in preventing SARS-CoV-2 infection in hematopoietic stem cell transplantation (HSCT) recipients during the early post-transplant period, a single-center, randomized controlled trial was conducted between June 2023 and February 2024. Seventy-two participants were randomized 1:1 to receive CCP (200 mL on days +14, +28, +2 months, and +3 months post-transplant) or standard treatment. The primary outcome was COVID-19 infection within 28 days after the last infusion; secondary outcomes included severe infection, adverse events, and survival. The CCP and standard treatment group each included 36 patients. In the CCP group, 26 (72.2%, 26/36) patients completed four cycles of plasma infusion and 33 (33/36, 91.7%) patients received at least one infusion. The median time to infection was 95 days (IQR 81-106) in the CCP group and 74 days (IQR 48-110) in the standard group (p = 0.272). The 120-day cumulative COVID-19 incidence was 32.1% (95% CI, 14.5-46.1%) in the CCP group and 22.9% (95% CI, 7.6-35.6%) in the standard treatment group (p=0.459). Severe cases occurred in one patient per group, with no 30-day mortality. One-year overall survival (OS) was lower in the CCP group compared to the standard treatment group (73.1% [95% CI, 59.4-90.0%] vs. 91.7% [83.1-100%], p=0.044). At 90 days post-transplantation, the peripheral blood CD4/CD8 ratio in patients of the CCP group was significantly lower than that in the standard treatment group (p = 0.008). Adverse events were observed in only 3 patients and were mild. CCP recipients had higher antibody levels, but differences were not significant. These findings indicate that CCP did not prevent COVID-19 infection and may have limited efficacy in early post-transplant patients.

**Clinical Trials:**

https://clinicaltrials.gov/, identifier NCT05904067.

## Introduction

The coronavirus disease 2019 (COVID-19) pandemic, caused by the severe acute respiratory syndrome coronavirus 2 (SARS-CoV-2), has become the most significant global public health crisis of the past century. By December 2024, over 777 million cases and 7 million deaths had been reported to the World Health Organization (WHO) (1).

Hematopoietic stem cell transplantation (HSCT) recipients are especially vulnerable to severe COVID-19 due to delayed immune reconstitution, which increases risks of prolonged viral shedding and elevated mortality rates (2). The early post-transplant period is particularly critical, as severe immunosuppression diminishes vaccine responses, with additional doses potentially insufficient or exacerbating graft-versus-host disease (GVHD) (3–6). While ursodeoxycholic acid (UDCA) was initially suggested to prevent SARS-CoV-2 infection, our research found no protective effects in this population (7). These challenges underscore the urgent need for alternative preventive strategies tailored to this high-risk group.

Convalescent plasma (CCP), derived from individuals who have recovered from COVID-19, offers a promising alternative. By providing passive immunization, CCP contains polyclonal antibodies that can neutralize SARS-CoV-2, inhibit viral replication, and potentially modulate the host immune response (8–12). The WHO recommends its use in clinical trials for severe and critical COVID-19 cases (13), a category that includes early post-transplant patients undergoing HSCT due to their profound immunosuppression. Although CCP has shown potential in reducing disease severity among HSCT recipients with COVID-19 (14–16), evidence regarding its role as a preventive measure, particularly in the early post-transplant period, remains limited and underexplored.

In this context, we conducted a randomized controlled trial to evaluate early passive transfer of high-titer anti–SARS-CoV-2 antibodies via CCP administered at four predefined time points (+14 days, +28 days, ~2 months, and ~3 months), which was aligned with the immunologic nadir, the gradual transition toward immune recovery, and clinical milestones such as protective-isolation discontinuation or discharge following engraftment (17, 18). This study reports one-year outcomes, providing critical insights into preventing COVID-19 in this highly immunocompromised population.

## Methods

### Trial design and setting

The *CCP-COVID-19-HSCT-2023* trial (ClinicalTrials.gov NCT05904067) was a prospective, single-center, randomized controlled trial conducted at the Hematopoietic Stem Cell Transplantation Center, Institute of Hematology & Blood Diseases Hospital, between June 2023 and February 2024. Participants were randomly assigned in a 1:1 ratio to the CCP group or standard treatment group using a centralized randomization system and were followed for 12 months. A detailed description of the study design, intervention schedule, and safety monitoring procedures can be found in the full protocol (**Supplementary Appendix 1**).

### Randomization and allocation concealment

The randomization sequence was computer-generated by an independent statistician using a simple randomization algorithm and was not accessible to the clinical investigators. Allocation concealment was maintained through sealed, sequentially numbered opaque envelopes prepared by the data manager, which were opened only after each participant had provided written informed consent and eligibility was confirmed.

This process ensured that investigators enrolling patients could not predict group assignment prior to randomization. The study was conducted in an open-label design. To minimize potential bias, laboratory personnel conducting SARS-CoV-2 PCR testing and antibody-titer assays were blinded to group allocation. In addition, statistical analyses were performed on a de-identified dataset with coded treatment groups until data lock. Investigators and participants were aware of treatment assignment only for clinical management and safety monitoring purposes.

### Blinding procedures

The trial was conducted in an open-label design, as blinding of plasma infusion was not feasible. To minimize potential bias, laboratory personnel conducting SARS-CoV-2 PCR testing and antibody-titer assays were blinded to group allocation. In addition, investigators and participants were aware of treatment assignment only for clinical management and safety monitoring purposes.

### Ethical approval

The trial protocol was approved by the Institutional Review Board (IIT2023001-EC-1). Written informed consent was obtained from all participants before enrollment. The study adhered to the Declaration of Helsinki and good clinical practice guidelines.

### Participants and eligibility criteria

Eligible participants were adults (≥16 years) undergoing HSCT. Exclusion criteria included a history of COVID-19, SARS-CoV-2 positivity within 13 days post-transplantation, known HIV, active HBV or HCV, psychiatric disorders, severe plasma protein allergies, or IgA deficiency. Detailed criteria are in the study protocol (**Supplementary Appendix 1**).

### Intervention

The CCP group received 200 mL of COVID-19 CCP (neutralizing antibody titer ≥1:160) on days +14, +28, +2 months, and +3 months post-transplantation, alongside standard treatment (see **Supplementary Table 1** for detailed references and pathway mapping). CCP was sourced from COVID-19-recovered donors (negative nucleic acid tests) within two weeks, as fresh or frozen plasma.

### Antibody assessment

Anti-SARS-CoV-2 IgG in donor plasma was measured using the WANTAI SARS-CoV-2 IgG ELISA kit (Beijing Wantai Biological Pharmacy Enterprise Co., Ltd., China), following the manufacturer’s instructions. This assay provides semiquantitative signal-to-cutoff (S/CO) ratios and is intended for qualitative determination of antibody positivity. According to the standards used by national blood centers in China, plasma with a neutralizing antibody titer ≥1:160 was defined as high-titer CCP. The assay is not calibrated to the WHO International Standard, and conversion to binding antibody units per milliliter (BAU/mL) was therefore not feasible. All antibody data in this study are thus reported as semiquantitative results based on the manufacturer’s criteria (details in **Supplementary Material**).

### Concomitant medications and standard supportive care

Both study arms received identical post-transplant supportive care, including antimicrobial prophylaxis, GVHD prevention, and hepatoprotective therapy. UDCA was routinely administered to all participants (250 mg three times daily) according to institutional protocol to prevent hepatic injury and cholestasis. UDCA was not considered part of the study intervention, and its use and dosage were consistent across both groups. Previous evidence from our institution confirmed that UDCA has no antiviral or anti–SARS-CoV-2 effect (7).

### Safety assessments

Adverse events were graded using CTCAE v5.0 and EBMT guidelines, with toxicity relationships categorized as certain, probable, possible, or unlikely per WHO criteria.

### Data collection and outcomes

Data were collected via electronic medical records. Participants underwent regular SARS-CoV-2 nucleic acid and antibody testing. Severe cases prompted chest CT, liver and kidney function tests, and arterial blood gas analysis for oxygen saturation <94%.

The primary outcome was the incidence of laboratory-confirmed COVID-19 infection from the first CCP infusion to 28 days after the last infusion, corresponding approximately to day +120 after transplantation. This window covers the entire prophylactic period of CCP administration (days +14, +28, +60, and +90). Secondary outcomes included severe infections, 30-day survival post-infection, one-year OS, and adverse reactions to CCP (definitions in **Supplementary Material**). Outcomes align with the 120-day post-transplantation timeframe for data presentation.

### Statistical analysis

A pre-specified analysis plan included ITT analyses to ensure comprehensive evaluation. The sample size was determined based on the expected difference in COVID-19 infection rates between the two study arms. The infection rate within one year after HSCT was estimated at 80% in the control group and 50% in the CCP group, based on preliminary institutional data and published reports. Using a two-sided significance level (α = 0.05) and a power of 80% (β = 0.20), and applying a log-rank test for group comparison, the required total sample size was calculated to be 72 participants (36 per group). A 10% attrition rate was incorporated to account for potential losses to follow-up. The calculation was performed using PASS version 15.0 (NCSS, Kaysville, UT, USA). The sample size and methods are detailed in the **Supplementary Appendix 1**. Continuous variables were compared using the Mann–Whitney U-test, and categorical variables using Chi-squared test or Fisher’s exact test. Kaplan–Meier plots and log-rank tests were used for time-to-event analyses. To evaluate the 120-day cumulative infection rate, we used Royston-Parmar flexible parametric survival models, adjusting for confounders (age, sex, and HCT-CI scores). All results are reported with corresponding 95% confidence intervals (CIs) and p values. Statistical significance was defined as a two-sided p < 0.05 after correction, unless otherwise specified. Statistical analyses were performed using R software, version 4.1.3, and SPSS 20.

## Results

### Patients

**Figure 1:**
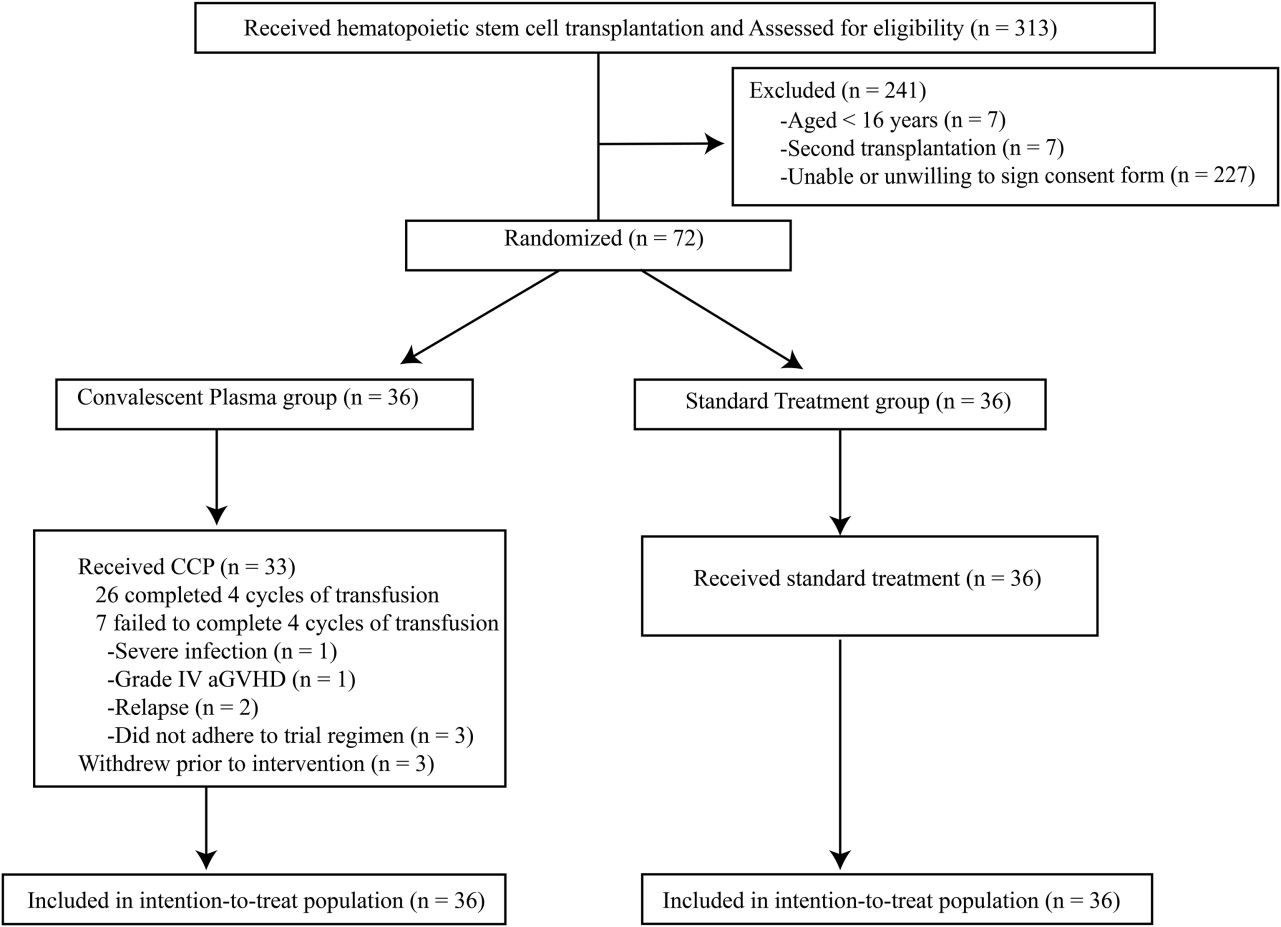
Participant CONSORT diagram. Baseline SARS-CoV-2 status was determined using serology or respiratory swab PCR testing. PCR, polymerase chain reaction.

Between June 2023 and February 2024, 313 patients undergoing HSCT were screened, with 72 randomized 1:1 to the standard treatment group (n=36) or the CCP group (n=36) (**Figure 1**). Three patients from the CCP group withdrew before receiving any infusion, resulting in an overall withdrawal rate of 4.2%. Among the 33 CCP recipients, 26 (26/33, 78.8%) completed all four cycles. Infusion was discontinued in 4 (4/33, 12.1%) patients due to severe aGVHD, pulmonary infection, or hematological relapse, and three (3/33, 9.1%) missed scheduled infusions due to non-adherence. In this study, 72 patients were analyzed under intention-to-treat (ITT) principles. The baseline characteristics were shown in **Table 1**.

**Table 1:** Characteristics at baseline of patients in the intention-to-treat (ITT) analysis.

Characteristics	Standard group (n = 36)	CCP group (n = 36)	p value
Age (median [IQR])	43 (37, 50)	39 (23, 50)	0.269
Gender			
male	19 (52.8)	19 (52.8)	1
female	17 (47.2)	17 (47.2)	
Disease			
AML	20 (55.6)	17 (47.2)	0.346
ALL	5 (13.9)	11 (30.6)	
MDS	4 (11.1)	4 (11.1)	
Other	7 (19.4)	4 (11.1)	
Disease type			1
Malignant	36 (100.0)	35 (97.2)	
Non-malignant	0	1 (2.8)	
Transplantation type			0.750
HIDT	8 (22.2)	10 (27.8)	
MSDT	26 (72.2)	25 (69.4)	
MUDT	2 (5.6)	1 (2.8)	
COVID-19 infection before transplantation			1
no	31 (86.1)	30 (83.3)	
yes	5 (13.9)	6 (16.7)	
HCT-CI scores			1
0-1	32 (88.9)	33 (91.7)	
≥ 2	4 (11.1)	3 (8.3)	
Conditioning regimen			0.414
BUCY-based	29 (80.6)	25 (69.4)	
TBI-based	7 (19.4)	11 (30.6)	
GVHD prophylaxis			0.198
CNI+MTX	5 (13.9)	3 (8.3)	
CNI+MTX+MMF+ATG	30 (83.3)	28 (77.8)	
CNI+MTX+ATG	1 (2.8)	5 (13.9)	

HIDT, haploidentical donor transplantation; MSDT, matched sibling donor transplantation; MUDT, matched unrelated donor transplantation; HCT-CI, hematopoietic cell transplantation-specific comorbidity index; BU, busulfan; CY, cyclophosphamide; TBI, total body irradiation; CNI, calcineurin inhibitor; MTX, methotrexate; MMF, mycophenolate mofetil; ATG, anti-thymocyte globulin.

### Primary outcome

**Figure 2:**
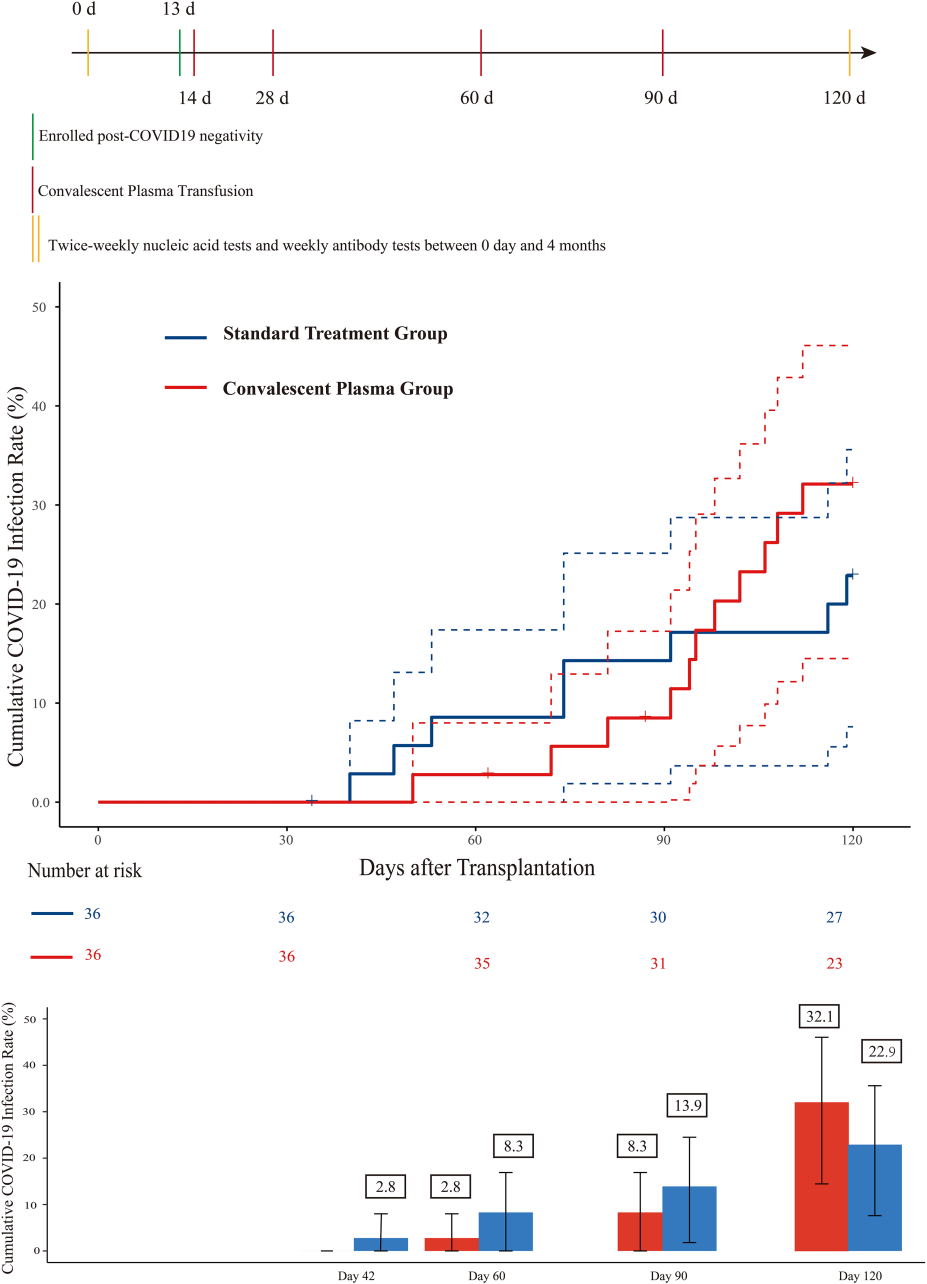
Study timeline, cumulative infection rate, and subgroup analysis of COVID-19 incidence after HSCT. Study timeline and monitoring schedule. Eligible HSCT recipients who tested negative for SARS-CoV-2 nucleic acid after transplantation were enrolled (green line, day +13). High-titer COVID-19 convalescent plasma (CCP) was administered prophylactically at four predefined time points: day +14, day +28, approximately day +60, and day +90 (red lines). From day 0 to day +120, participants underwent twice-weekly nucleic acid testing and weekly antibody testing (yellow markers) to monitor viral status and antibody kinetics. Cumulative incidence curves of laboratory-confirmed COVID-19 infection within 120 days post-transplantation. The red line represents the CCP group, and the blue line represents the standard treatment group. Dashed lines indicate 95% confidence intervals. The CCP group demonstrated a numerically lower infection rate compared with the standard treatment group. Bar chart showing cumulative infection rates at days 42, 60, 90, and 120 post-transplantation. CCP group: 0% at day 42, 2.8% (0-8.0%) at day 60, 8.3% (0-17.2%) at day 90, and 32.1% (14.5-46.1%) at day 120; control group: 2.8% (0-8.2%), 8.3% (0-17.4%), 13.9% (3.7-28.7%), and 22.9% (7.6-35.6%) at the same time-points.

19 developed confirmed Omicron variant COVID-19 within 120 days post-transplant in this study. The median time from transplantation to infection occurrence was 95 (IQR, 81-106) days in the CCP group and 74 (IQR, 48-110) days in the standard treatment group (p = 0.272). Kaplan-Meier estimates of the 120-day cumulative incidence of infection showed no statistically significant difference between the CCP and standard groups (32.1% [95% CI, 14.5-46.1%] vs. 22.9% [95% CI, 7.6-35.6%]; p=0.459) (**Figure 2**). Despite numerically lower infection rates in the CCP group at early timepoints, no statistically significant protection was observed through 120 days (**Figure 2**). Besides, adjusted estimated risks at 120 days were 26.4% (95% CI, 12-40.8%) for the CCP group and 18.1% (95% CI, 5.5-30.7%) for the standard group (Difference, 8.3% [95% CI, -10.8%-27.4%]; p = 0.400; **Supplementary Figure 1A**). The 120-day cumulative incidence of infection was analyzed according to the number of plasma infusion cycles. There was no significant difference among patients who received four cycles of infusion, fewer than four cycles, or standard treatment (*p* = 0.365; **Supplementary Figure 2**).

### Secondary outcomes

**Figure 3:**
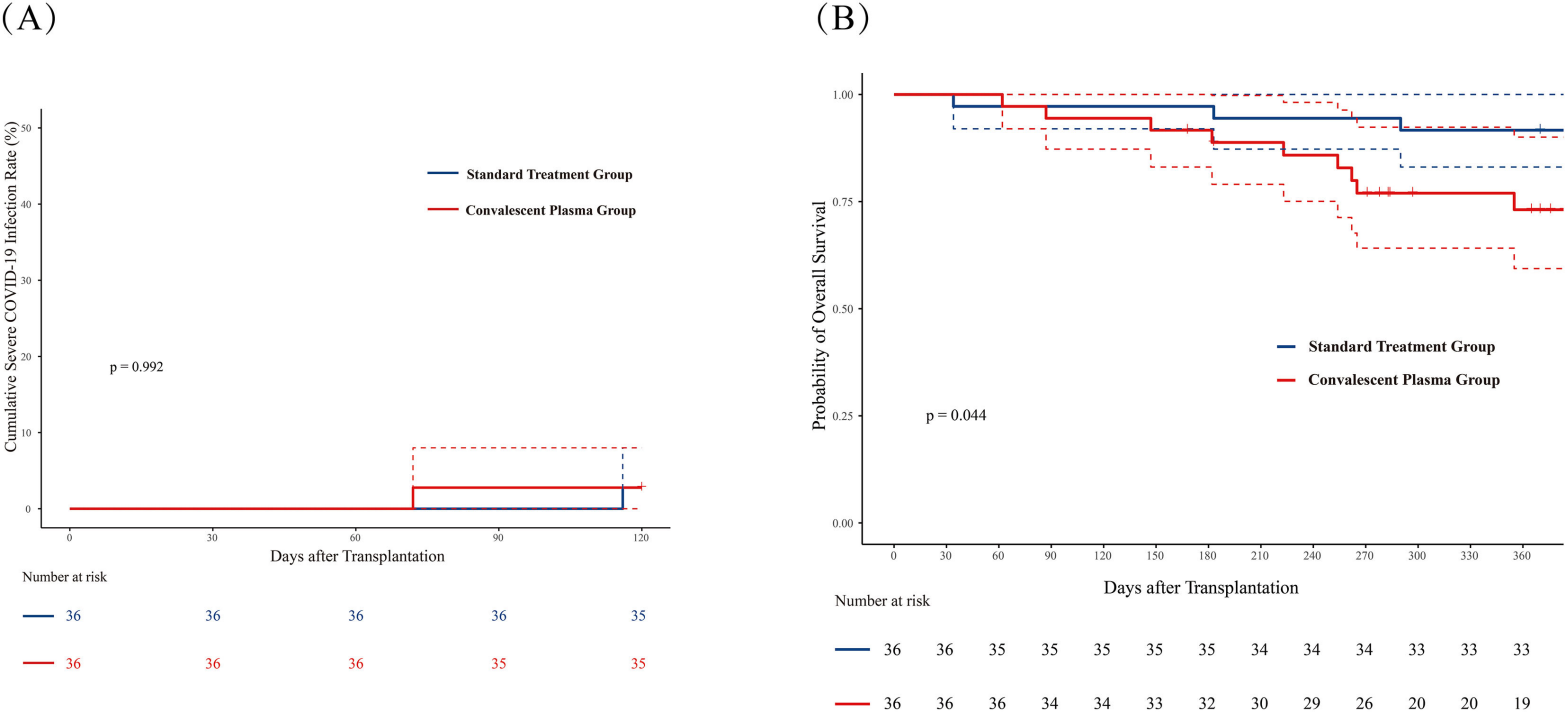
The cumulative incidence of severe COVID-19 infection and overall survival (OS) at 365 days post-HSCT. **(A)** Cumulative incidence of severe COVID-19 infection within 120 days post-transplantation. **(B)** One-year overall survival after HSCT. The convalescent plasma (CCP) group (red) and standard treatment group (blue) were compared using the log-rank test. The red line represents the CCP group, and the blue line represents the standard treatment group. Dashed lines indicate 95% confidence intervals.

Two patients developed severe COVID-19 infection, with one case in each group [2.8% (95%CI, 0-8%)] for both CCP and control groups. In the CCP group, the event occurred at 72 days post-transplantation after 3 infusions, whereas the control group case occurred at 116 days. The detailed information of patients in the CCP group was shown in **Supplementary Table 2**. The Kaplan-Meier estimated cumulative incidence of severe COVID-19 did not differ significantly between group (**Figure 3A**; p = 0.992). Neither of the two patients died within 30 days of infection.

During the follow-up period, a total of 12 (12/72, 16.7%) patients died, with 9 deaths (9/36, 25.0%) occurring in the CCP group and 3 deaths (3/36, 8.3%) in the standard group. The detailed causes of death were shown in **Table 2**. The one-year OS rate was significantly lower in the CCP group compared to the standard group (73.1% [95% CI, 59.4-90.0%] vs. 91.7% [83.1-100%]; p=0.044, **Figure 3B**).

**Table 2:** Cause of death in the ITT populations.

Cause of Death	CCP group (n = 36)	Standard group (n = 36)
Relapse	5 (55.6)	0
Infection	4 (44.4)	1 (33.3)
GVHD	0	1 (33.3)
TATMA	0	1 (33.3)
Total	9 (100.0)	3 (100.0)

GVHD, graft versus host disease; TATMA, Transplantation-Associated Thrombotic Microangiopathy.

To assess whether the number of plasma infusion cycles affected survival, we performed a stratified analysis. In the CCP group, patients who received four cycles had a 1-year OS of 79.2% (95% CI, 64.4-97.4%), comparable to the standard treatment group [91.7% (95% CI, 83.1-100%); *p* = 0.182]. In contrast, patients who received fewer than four cycles had a significantly lower 1-year OS of 57.1% (95% CI, 32.6-100%; p = 0.013) (**Supplementary Figure 2**). Among the 10 patients who did not complete the full course of plasma infusion, 4 died, including 2 from disease relapse, 1 from severe pneumonia unrelated to COVID-19, and 1 from severe intestinal infection and GVHD.

Among the CCP group, 5 patients (5/36, 13.9%) suffered hematological relapsed, while in the standard group, only 2 patients experienced extramedullary relapse (2/36, 5.6%) within one year post-transplantation. Of the 5 patients in the CCP group, 3 completed 4 cycles of plasma infusion, with relapse occurring at 135, 176, and 262 days post-transplantation, respectively. One received 2 cycles of plasma infusions and relapsed at 57 days post-transplantation. The remaining one did not receive any plasma infusions and relapsed at 122 days post-transplantation.

**Figure 4:**
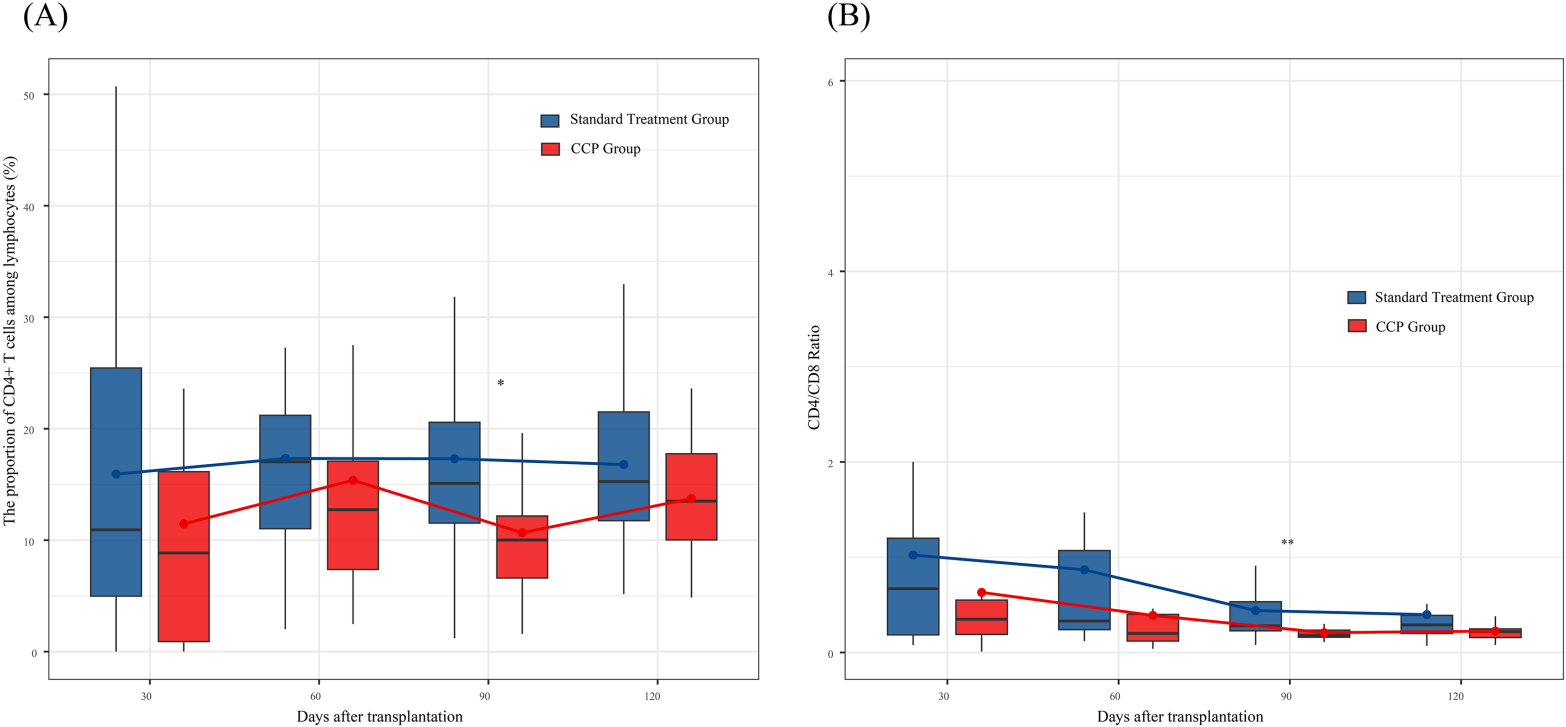
CD4^+^T-cell immune reconstitution after transplantation within 120 days. **(A)** The distribution of peripheral blood CD4+ T-cell frequency (%); **(B)** The ratio of CD4+ T cell to CD8+ T cell.Boxes represent the first and third quartiles; lines inside boxes represent median values, with whiskers extending to 1.5 times the interquartile range (IQR) from the quartiles; circles inside boxes represent mean values. The Mann-Whitney U test was used for between-group comparisons; *p < 0.05; **p < 0.01.

Post-transplant immune reconstitution analysis was performed. Overall, there were no significant differences in the proportions of T cells, B cells, and NK cells in the peripheral blood (**Supplementary Figure 3**). However, at 90 days post-transplantation, the proportion of CD4+ T cells among lymphocytes and the CD4/CD8 ratio in the CCP group were significantly lower than those in the standard group (**Figure 4**; p = 0.012 and p = 0.008, respectively).

### Adverse events

Among the 33 patients in the CCP group who received at least one infusion, adverse events were observed in 3 cases (3/33, 9.1%). The most common adverse event was fever, reported in 2 patients (2/33, 6.1%), followed by rash in 1 patient (1/33, 3.0%). All events resolved within 24 hours with supportive care, and no serious adverse events were reported (**Table 3**; **Supplementary Table 3**).

**Table 3:** Adverse Events in patients receiving convalescent plasma (n=33).

AEs	Number of cases (n)
Fever	2 (66.7)
Rash	1 (33.3)
Chills	0
Itching	0
Hypotension	0
Dyspnea	0
Liver Dysfunction	0
Heart Failure	0
Renal Failure	0
shock	0
Total	3 (100.0)

### Antibody dynamics

All plasma units used for infusion met the national criterion for high-titer convalescent plasma (≥1:160 by ELISA). Antibody absorbance (optical density, OD) and neutralizing titers were measured at predefined intervals post-HSCT. Antibody testing was semiquantitative and not directly comparable to WHO-standardized neutralizing titers. Median absorbance levels were consistently higher in the CCP group at Days 28, 60, and 90, but without statistical significance (**Supplementary Figure 4A**). OD levels were also lower in infected compared to non-infected patients up to Day 21, though the differences were not significant (**Supplementary Figure 4B, C**).

Neutralizing antibody seropositivity (titer ≥1:160) remained low across groups. Further details were available in **Supplementary Table 4**. The cumulative infection rate at 120 days post-transplant was significantly lower in antibody-positive patients than in antibody-negative patients (14.0% [95% CI, 1.8-24.7%] vs. 41.2% [95%CI, 22.1-55.6%]; p = 0.016). In addition, antibody titer levels had no significant impact on 1-year OS (83.4% [95% CI, 72.1-96.5] for antibody-positive vs. 82.1% [95% CI, 70.1-96.2] for antibody-negative; *p* = 0.896; **Supplementary Figure 2**).

### Subgroup analysis

To evaluate the efficacy of CCP across different subgroups, we performed analyses stratified by age, gender, type of transplant, and HCT-CI scores for the 120-day cumulative infection rate (**Supplementary Figure 5**). No significant interactions were observed in any subgroup. However, a non-significant trend toward better efficacy was noted in females compared to males (p = 0.089), warranting further investigation.

## Discussion

In this single-center randomized controlled trial evaluating patients with incomplete immune reconstitution early after HSCT, CCP administration showed a trend toward delaying the time to COVID-19 infection but did not significantly reduce the cumulative incidence within one month after the last CCP infusion. One-year OS was also lower in the CCP group, indicating its limited prophylactic effect.

Although the WHO declared the end of the global COVID-19 emergency in May 2023, SARS-CoV-2 remains prevalent in communities and healthcare settings (19, 20). COVID-19 in HSCT patients is often symptomatic and associated with significantly higher mortality compared to the general population (21). Notably, the risk of moderate to severe COVID-19 is highest within the first 100 days post-allogeneic HSCT, with an incidence of 34.14%, which decreases to 18.90% between 100 days and 1 year, and further to 3.68% between 1 and 2 years post-transplantation (22). This elevated early risk underscores the importance of implementing effective measures to reduce COVID-19 infection, particularly during the critical early post-transplantation period.

CCP from recovered COVID-19 patients contains polyclonal antibodies that neutralize SARS-CoV-2, reduce viral load, secondary infections, and inflammation, with low adverse reaction rates, making it a therapeutic option for immunodeficient hematological malignancy patients with COVID-19 (23, 24). A cohort study of 966 hematological malignancy patients found lower 30-day mortality in CCP recipients than controls (13.3% vs. 24.8%) (24), and Hueso et al. reported reduced mortality in B-cell malignancy patients with prior anti-CD20 therapy (25). However, another multicenter retrospective study involving 313 hematological malignancy patients with COVID-19 (80.2% had lymphocytic malignancies) did not observe the clinical efficacy of CCP (P>0.100) (26). In the transplantation field, individual case reports provide additional insights. One lymphoma patient with prior autologous transplantation experienced prolonged SARS-CoV-2 positivity despite CCP (27), while four patients with persistent RNA positivity eventually responded to antiviral therapy after CCP failure (28). Despite variability in therapeutic efficacy, CCP is considered safe and well-tolerated, but its prophylactic role in immunocompromised populations, such as HSCT recipients, remains largely unexplored.

Our study contributes to the limited evidence on CCP’s preventive use in this population, particularly during the early post-transplant period. While CCP delayed the median time to infection, it did not significantly lower the overall COVID-19 incidence (32.1% vs. 22.9%; p=0.459). Meanwhile, our observation of a significantly reduced one-year OS (73.1% vs. 91.7%; p=0.044) in the CCP group suggests potential risks associated with this intervention in HSCT recipients. Subgroup analyses further confirmed its limited efficacy in reducing infection risk across clinical and demographic subgroups. These findings indicate that CCP neither prevented COVID-19 infection nor improved survival outcomes and, instead, was associated with a reduced one-year OS rate.

Recent randomized trials have likewise reported no significant benefit of CCP for COVID-19. Large platform studies such as RECOVERY (29) and REMAP-CAP (30), and multicenter RCTs including CONCOR-1 (31), DAWn-Plasma (32), PLACID (33), and the Argentinian NEJM trial (34), all failed to demonstrate improvement in survival or disease progression despite the use of high-titer plasma. A phase 2 prophylaxis trial (35) also found no preventive efficacy, though safety was confirmed. These results suggest that CCP confers limited benefit outside narrowly defined contexts involving early administration to seronegative hosts with high-functional antibody content. Our findings align with this broader evidence, extending it to the early post-HSCT prophylaxis setting characterized by profound lymphopenia and hypogammaglobulinemia.

Research suggests a correlation between SARS-CoV-2-specific IgG titers and neutralizing capacity (9, 23, 36). The WHO emphasizes the importance of neutralizing antibody titers in CCP efficacy and recommends reporting these titers and infusion volumes in clinical studies (13). A study on hospitalized COVID-19 patients demonstrated that infusion of CCP with high antibody levels was associated with reduced mortality (37). Similarly, a randomized trial found that early CCP administration (within 14 days of symptoms) improved viral clearance (9). In HSCT recipients, the prolonged immune reconstitution process presents a unique challenge, particularly delayed B-cell recovery in allogeneic transplant patients. B cells are essential for generating effective antibody responses, and their delayed recovery significantly impairs the ability to mount a robust humoral immune response. Our prior study revealed that median B-cell counts in these recipients remained below normal levels for up to 180 days post-transplantation (17). In our study, the CCP group showed slightly higher antibody absorbance levels than the standard treatment group but remained below the normal range between days 42 and 120 post-transplantation. This modest increase delayed the time to SARS-CoV-2 infection but failed to reduce the cumulative incidence of new infections. It is possible that elevated antibody levels negatively impacted humoral immune reconstitution, limiting the generation of effective endogenous antibodies and contributing to a higher infection rate of COVID-19 and reduced one-year OS. This delayed adaptive immune recovery likely contributed to the CCP group’s limited efficacy as a preventive strategy, as it hindered the development of effective and sustained antibody responses.

While CCP showed limited efficacy in preventing severe COVID-19 in early post-transplant patients, our findings affirm its safety in this vulnerable population. Among 36 CCP recipients, only 3 experienced mild fever or rash, which resolved quickly with antihistamines, and no severe adverse reactions were observed. These results are consistent with previous studies: Shen et al. reported only mild discomfort in critically ill COVID-19 patients treated with CCP (8), and Duan et al. documented no significant infusion-related reactions in severe cases (38). Collectively, these findings underscore CCP’s favorable safety profile, even in patients with severely compromised immunity.

Among the 19 patients with COVID-19 in our study, only 2 developed severe infections. Notably, both severe cases were complicated by secondary bacterial and fungal infections, which significantly worsened their clinical conditions and contributed to adverse outcomes. A multi-country study reported patients with bacterial infections were more likely to require intensive care (60.0% vs. 28.5%, p < 0.0001) and mechanical ventilation (47.6% vs. 15.6%, p < 0.0001) compared to those without clinically relevant pathogens. In-hospital mortality was significantly higher in co-infected patients (31.7% vs. 13.2%, p < 0.0001) (39). These findings underscore the critical need for heightened surveillance and prompt management of co-infections in transplant recipients with COVID-19. Besides, previous studies showing a significant link between COVID-19 within the first 12 months post-HSCT and increased mortality (40, 41). The trend may reflect the compounded impact of COVID-19 on the already prolonged and incomplete immune reconstitution in allogeneic transplant recipients, where the infection, even if not directly fatal, further impairs recovery and contributes to reduced overall survival during the first-year post-transplant.

This study has certain limitations. The relatively small sample size limits the generalizability of our findings, although it was determined *a priori* through power calculation to ensure adequate statistical validity for the primary endpoint. While the CCP used in this study was standardized with a neutralizing antibody titer ≥1:160 to ensure baseline efficacy, we did not investigate whether higher antibody titers or earlier administration might lead to improved clinical outcomes. Additionally, the evolving nature of SARS-CoV-2—including the emergence of new variants—may have influenced the effectiveness of CCP during the study period. Despite our efforts to minimize bias through blinded laboratory and statistical analyses, the open-label design may still have introduced subtle observer bias at the clinical level.

The relatively small sample size limits the generalizability of our findings. Although it was determined *a priori* by formal power calculation to provide 80% power at a two-sided α of 0.05 based on the expected difference in infection rates between groups, the study was adequately powered for its predefined primary endpoint. However, the modest cohort size resulted in wide confidence intervals for some secondary outcomes, limiting the precision of these estimates. Despite our best efforts to maintain blinding through blinded laboratory, the open-label design could still introduce minimal observer bias at the clinical level. While the CCP used in this study was standardized with a neutralizing antibody titer ≥1:160 to ensure baseline efficacy, antibody assessment was based on a semiquantitative ELISA rather than a fully quantitative neutralization assay. This limitation precluded precise comparison of antibody levels across plasma units or correlation with functional neutralizing activity. We did not evaluate whether higher antibody titers might lead to improved clinical outcomes. Additionally, the evolving nature of SARS-CoV-2—including the emergence of new variants—may have influenced the effectiveness of CCP during the study period.

In conclusion, regular administration of CCP during the early post-transplant period delayed the onset of COVID-19 infections but did not prevent infections, reduce the incidence of severe disease, or improve overall survival in HSCT recipients. Antibody levels in CCP recipients remained persistently low during the study period, potentially limiting the effectiveness of passive immunization. Future studies should focus on optimizing dosing strategies, exploring alternative immunomodulatory approaches, and evaluating the combined use of CCP with other preventive measures in this vulnerable population.

## Data Availability

The original contributions presented in the study are included in the article/**Supplementary Material**. Further inquiries can be directed to the corresponding authors.
