# Supplementary material for: Convalescent plasma therapy for COVID-19 prophylaxis in adults early post-hematopoietic stem cell transplantation: one-year outcomes from a randomized controlled trial
Source: Front Immunol. 2025 Nov 28;16:1626775. doi: 10.3389/fimmu.2025.1626775 (PMC12698628; doi:10.3389/fimmu.2025.1626775)
Supplement: Supplementary file 1 [file Table1.docx]

Supplementary Material

# Supplementary Table 1. Pathophysiological and clinical rationale for the four scheduled CCP infusions after HSCT, with key literature references.

| **Time point** | **Pathophysiological rationale** | **Clinical-pathway rationale** | **Key references** |
| --- | --- | --- | --- |
| **+14 days** | Period of deepest lymphopenia and hypogammaglobulinemia; absence of vaccine response; passive antibody transfer provides early protection. | Coincides with protective-isolation discontinuation or discharge after engraftment (ANC recovery), when exposure risk sharply increases. | Einarsdottir S *et al.*, *Blood Adv* 2022 [4]; Murray SM *et al.*, *Br J Haematol* 2022 [3]; EBMT Handbook 2024 (Ch. “Supportive Care”) [18]; Pavlů J *et al.*, *Bone Marrow Transplant* 2016 [19]. |
| **+28 days** | Antibody levels from first infusion begin to decline (IgG half-life ≈ 21 days); adaptive humoral immunity still delayed. | Early outpatient follow-up phase; continued vulnerability before immune recovery. | Vidarsson G *et al.*, *Front Immunol* 2014 [20]; EBMT Handbook 2024 (Ch. “Vaccination”) [22]. |
| **≈ 2 months** | Transition point (~day 60) where lymphocyte subsets start to recover but remain functionally immature. | Corresponds to intensive outpatient monitoring (≤ day +100). | Cao Y *et al.*, *Am J Hematol* 2023 [21]; EBMT Handbook 2024 (Ch. “Follow-up after HCT”) [22]. |
| **≈ 3 months** | Ongoing impaired vaccine immunogenicity and incomplete humoral reconstitution. | Marks the shift from intensive to monthly follow-up; early vaccine programs begin but responses remain suboptimal. | EBMT Handbook 2024 (Ch. “Vaccination and Long-Term Follow-up”) [22]; Shanbhag S *et al.*, *TCT* 2022 [17]; Ljungman P *et al.*, *TCT* 2021 [18]. |

# Supplementary Table 2. Detailed information of patients in the CCP group (n=36)

| Number | Gender/Age | HCT-CI | Numbers of transfusion | COVID-19 infection within 120 days | Infection classification | Death/Cause |  |
| --- | --- | --- | --- | --- | --- | --- | --- |
| 1 | F/56 | 3 | 4 | yes | severe | yes/pneumonia | |
| 2 | M/32 | 0 | 4 |  |  |  | |
| 3 | M/40 | 0 | 4 | yes | moderate |  | |
| 4 | F/59 | 0 | 4 | yes | moderate | yes/pneumonia | |
| 5 | F/19 | 0 | 4 |  |  |  | |
| 6 | F/60 | 0 | 4 | yes | moderate | yes/relapse | |
| 7 | M/24 | 0 | 4 |  |  |  | |
| 8 | F/18 | 0 | 4 |  |  |  | |
| 9 | M/32 | 0 | 4 |  | mild |  | |
| 10 | F/32 | 0 | 3 | yes | mild |  | |
| 11 | F/53 | 1 | 4 | yes | moderate |  | |
| 12 | M/34 | 0 | 4 |  |  |  | |
| 13 | M/38 | 3 | 0 |  |  |  | |
| 14 | M/50 | 1 | 0 | yes | moderate |  | |
| 15 | F/24 | 0 | 4 | yes | mild |  | |
| 16 | M/16 | 0 | 0 | yes | moderate | yes/relapse | |
| 17 | F/55 | 1 | 4 |  |  |  | |
| 18 | M/18 | 0 | 4 |  |  |  | |
| 19 | F/59 | 0 | 3 |  |  |  | |
| 20 | M/49 | 0 | 4 |  |  |  | |
| 21 | F/42 | 0 | 4 |  |  | yes/relapse | |
| 22 | M/39 | 1 | 3 | yes | moderate |  | |
| 23 | F/20 | 0 | 1 |  |  | yes/intestinal infection | |
| 24 | M/54 | 2 | 4 |  |  |  | |
| 25 | F/21 | 0 | 4 |  |  |  | |
| 26 | F/49 | 0 | 4 |  |  |  | |
| 27 | M/55 | 0 | 4 | yes | mild |  | |
| 28 | M/18 | 0 | 4 |  |  |  | |
| 29 | M/16 | 0 | 4 |  |  |  | |
| 30 | F/19 | 0 | 4 |  |  |  | |
| 31 | F/41 | 0 | 4 |  |  |  | |
| 32 | M/26 | 0 | 4 |  |  | yes/relapse | |
| 33 | M/38 | 0 | 4 |  |  |  | |
| 34 | F/51 | 0 | 2 |  |  | yes/relapse | |
| 35 | M/44 | 1 | 3 |  |  |  | |
| 36 | M/45 | 0 | 1 |  |  | yes/pneumonia | |

# Supplementary Table 3. Adverse events for each participant, graded by CTCAE v5.0

| **Patient ID** | **Group** | **Infusion No.** | **Adverse Event** | **CTCAE Grade** | **Outcome** |
| --- | --- | --- | --- | --- | --- |
| CCP-26 | CCP | 1 | Fever | 1 | Resolved |
| CCP-68 | CCP | 4 | Rash | 2 | Resolved |
| CCP-30 | CCP | 4 | Fever | 1 | Resolved |
| All others | — | — | No AEs | — | — |

# Supplementary Table 4. The variations of antibody titer after transplantation in Intention-to-Treat Population (n = 72)

| Number | group | Titer |
| --- | --- | --- |
| 1 | STP | 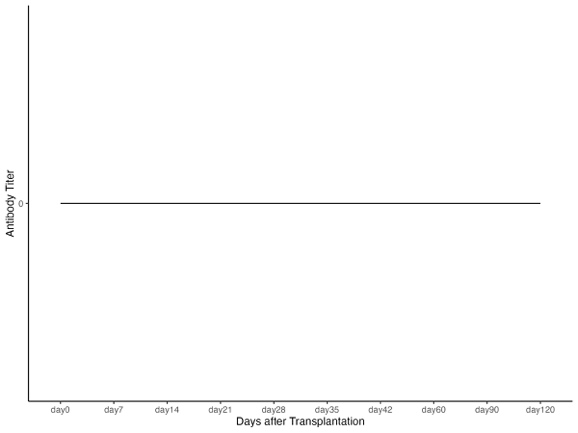 |
| 2 | CCP | 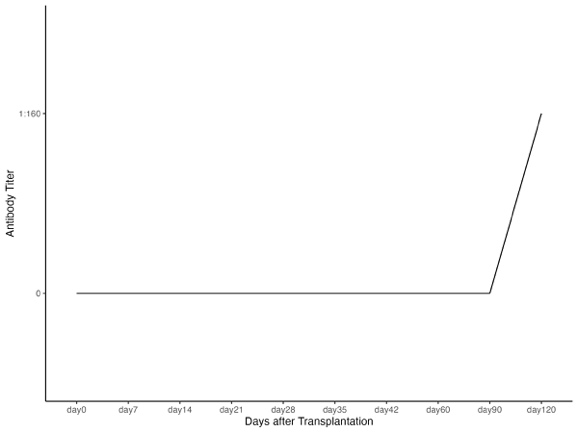 |
| 3 | STP | 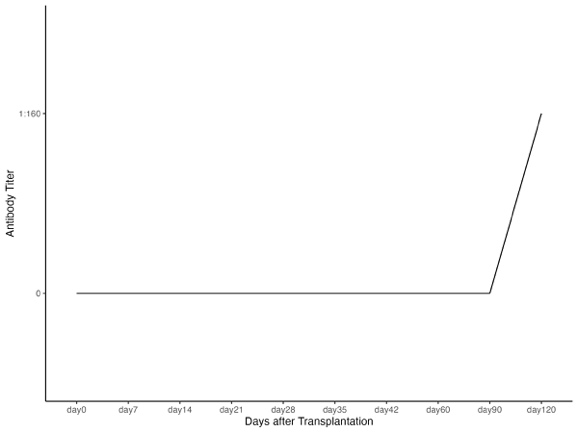 |
| 4 | CCP | 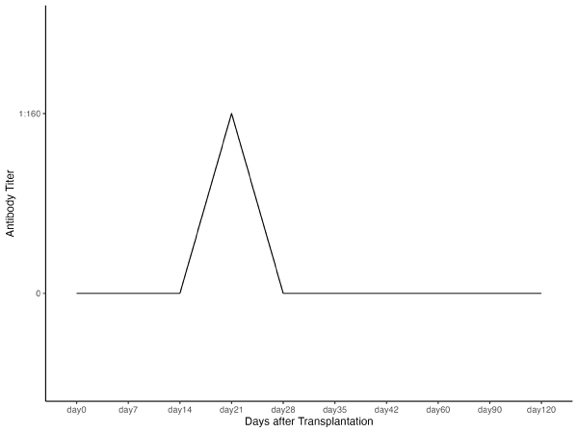 |
| 5 | CCP | 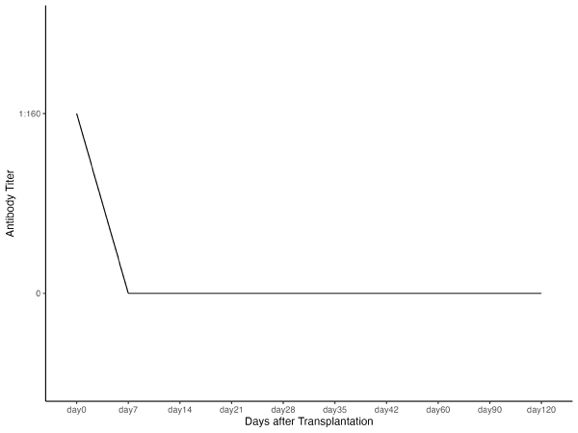 |
| 6 | CCP | 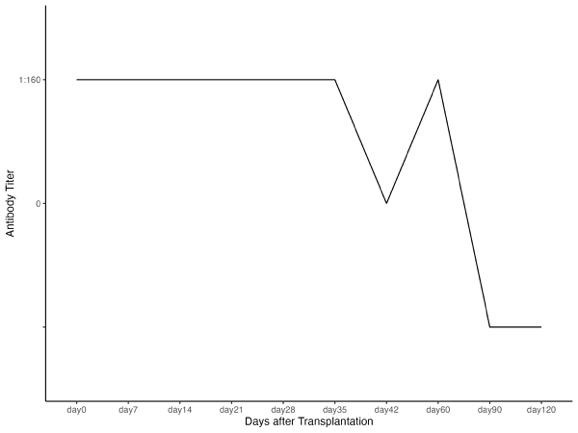 |
| 7 | STP | 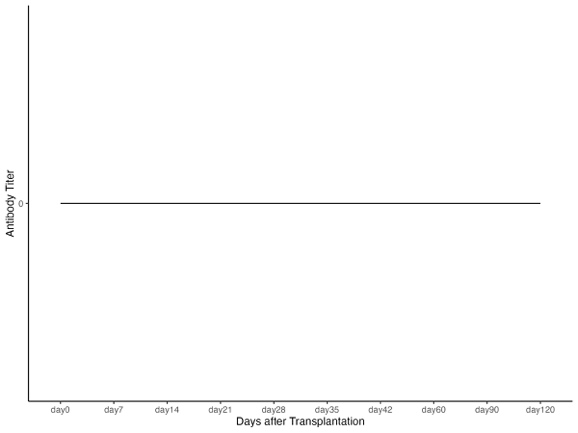 |
| 8 | STP | 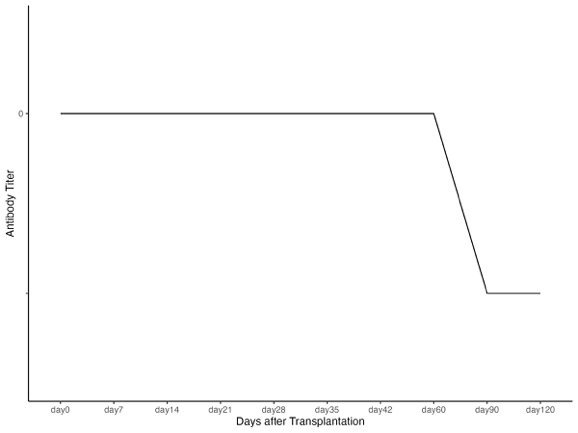 |
| 9 | CCP | 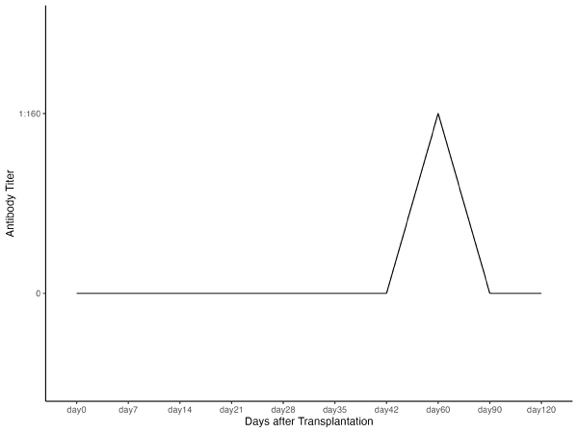 |
| 10 | CCP | 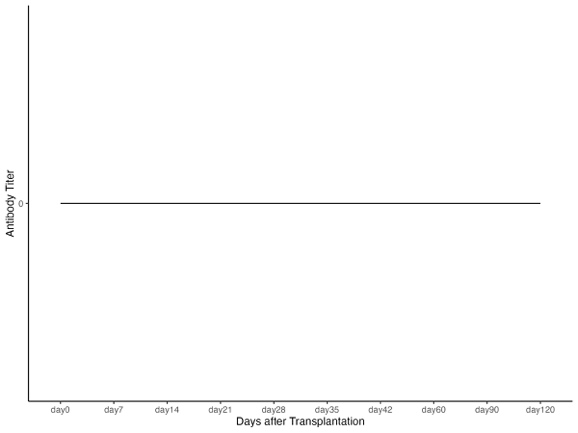 |
| 11 | CCP | 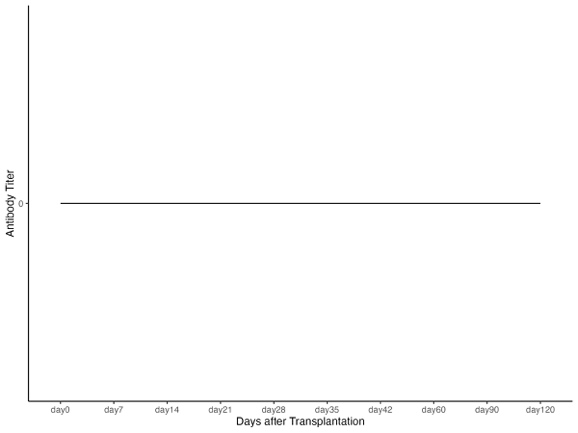 |
| 12 | STP | 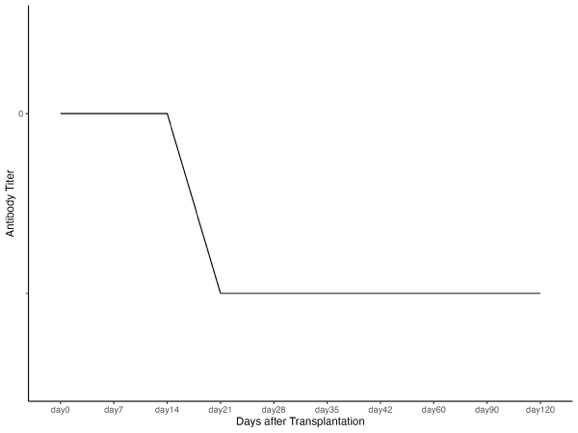 |
| 13 | CCP | 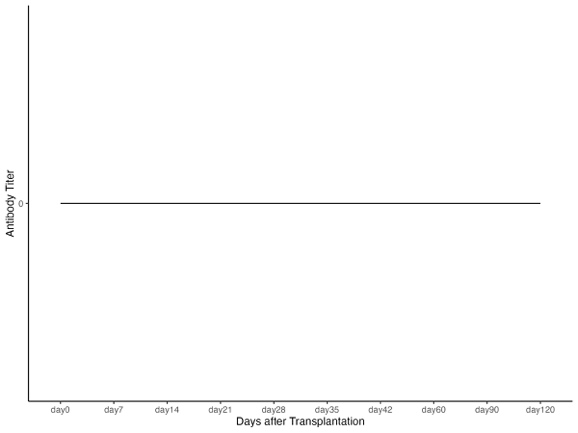 |
| 14 | CCP | 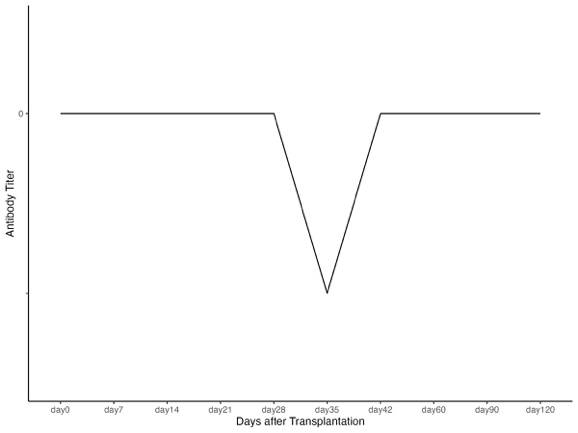 |
| 15 | STP | 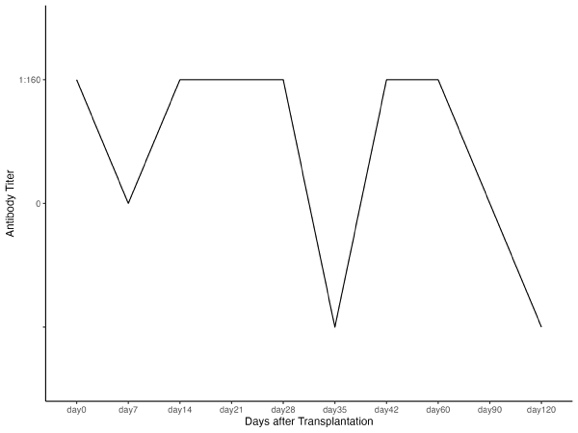 |
| 16 | STP | 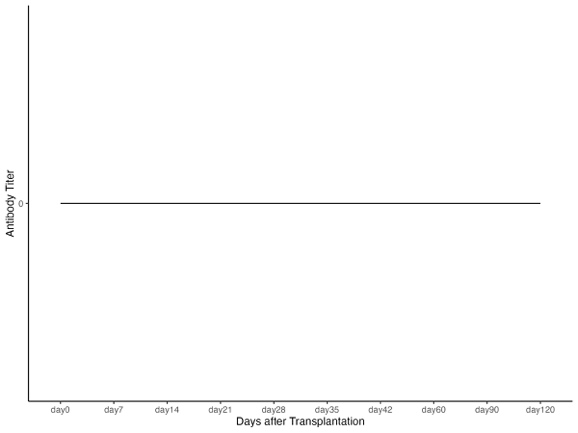 |
| 17 | STP | 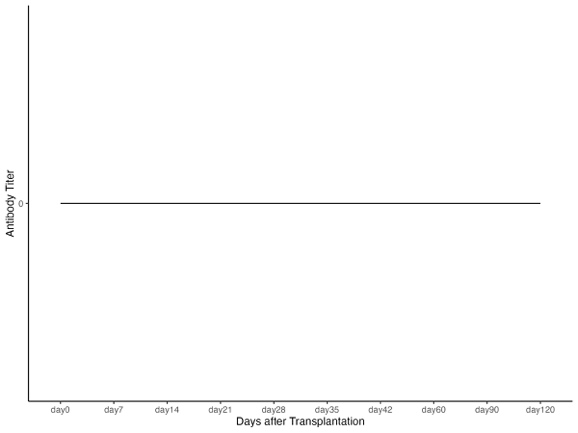 |
| 18 | STP | 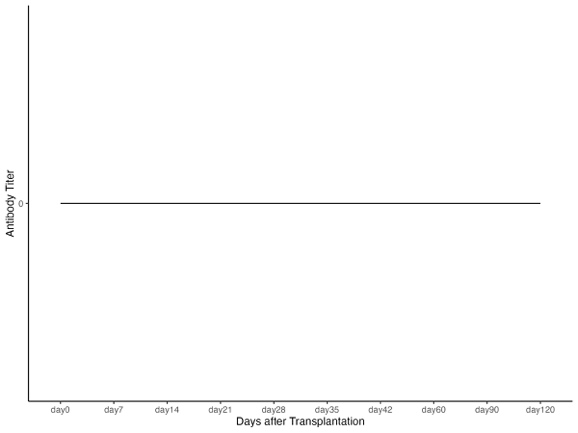 |
| 19 | CCP | 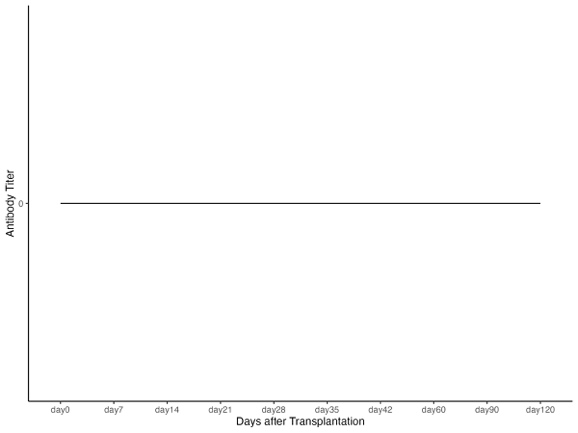 |
| 20 | STP | 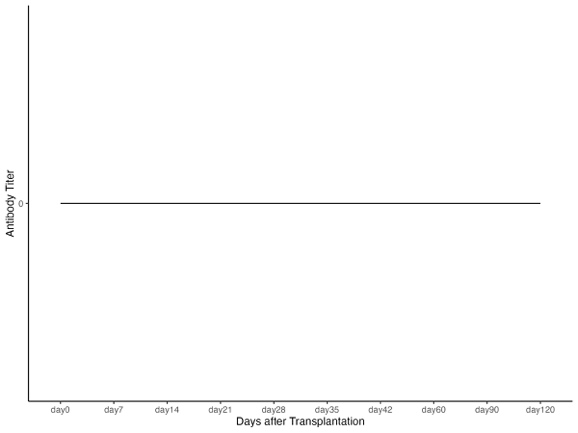 |
| 21 | STP | 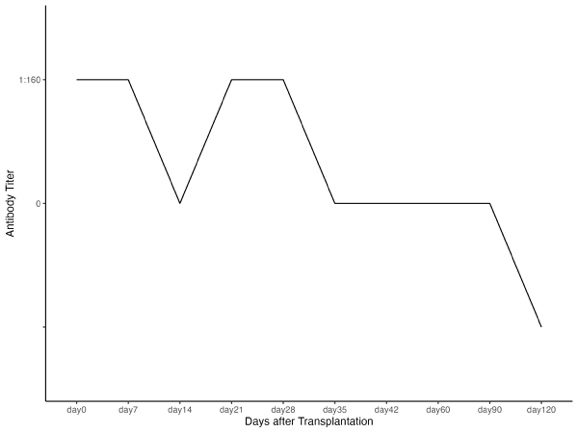 |
| 22 | CCP | 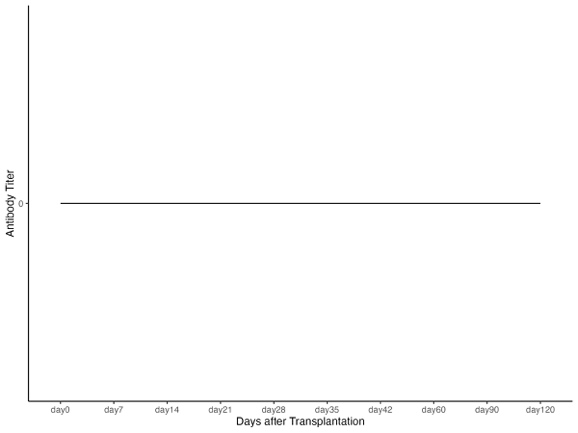 |
| 23 | STP | 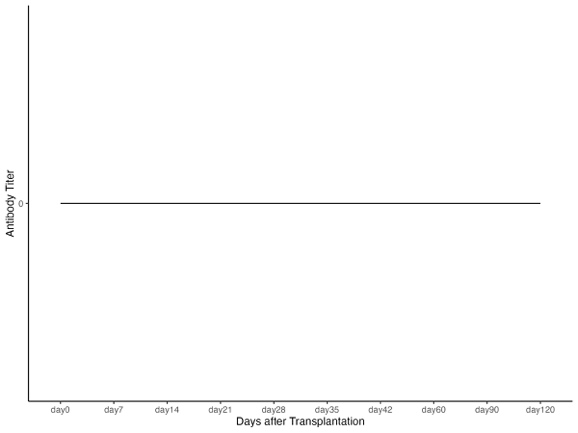 |
| 24 | STP | 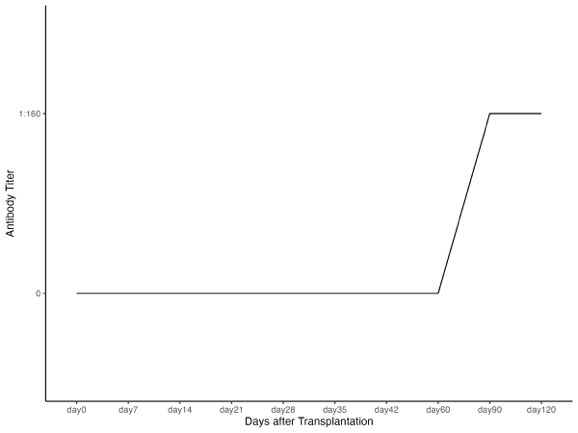 |
| 25 | STP | 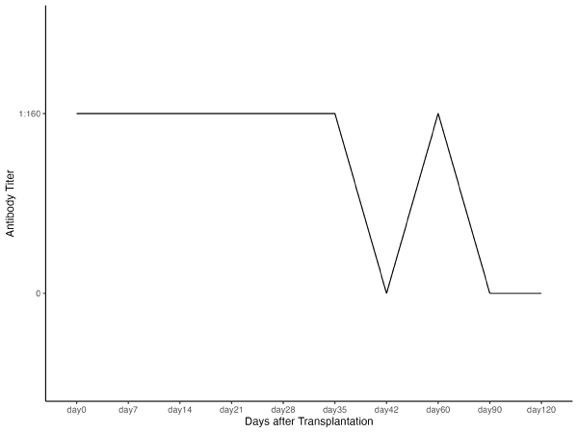 |
| 26 | STP | 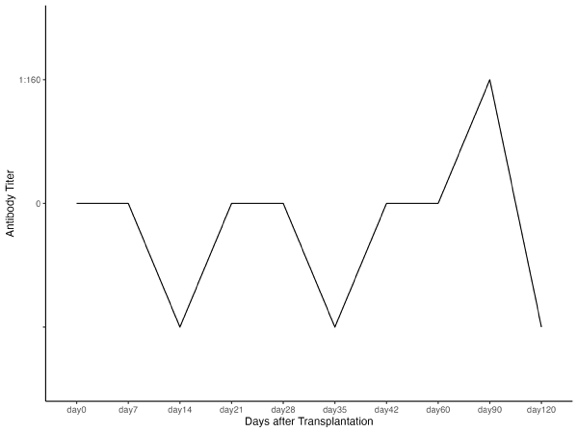 |
| 27 | STP | 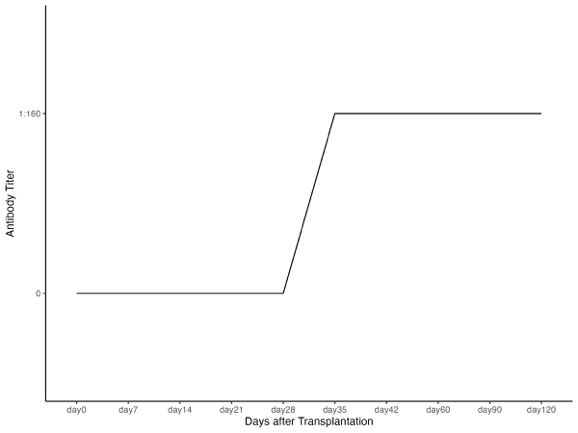 |
| 28 | CCP | 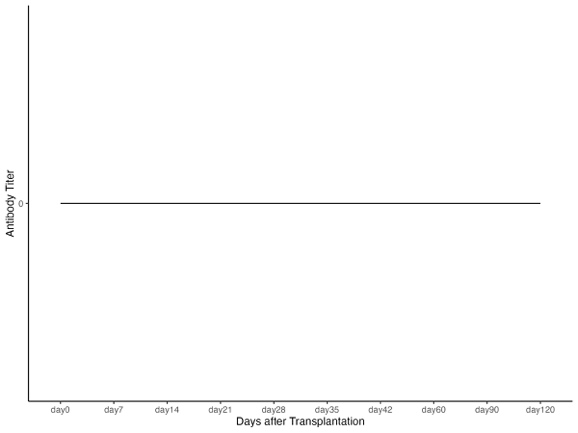 |
| 29 | STP | 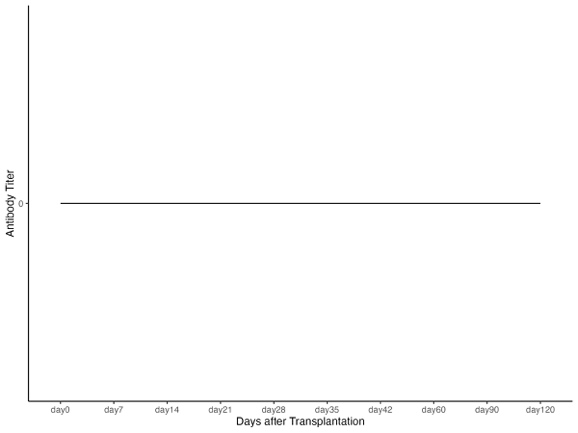 |
| 30 | STP | 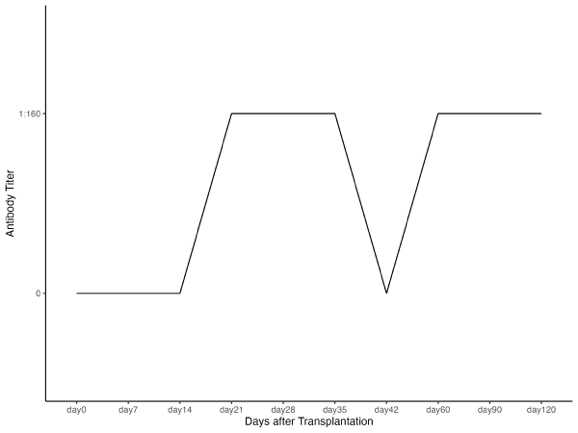 |
| 31 | CCP | 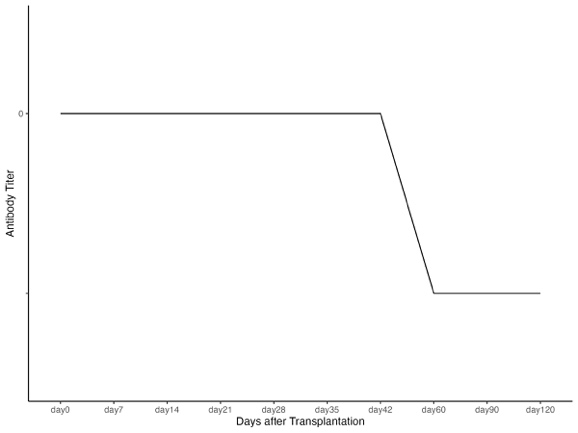 |
| 32 | CCP | 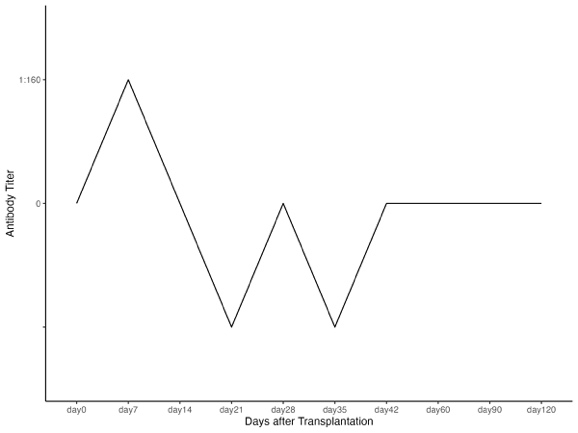 |
| 33 | STP | 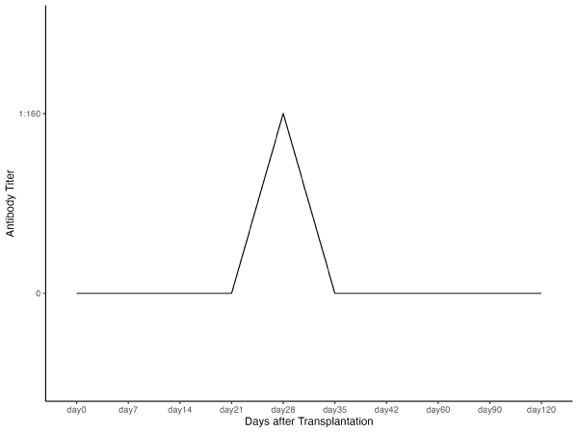 |
| 34 | CCP | 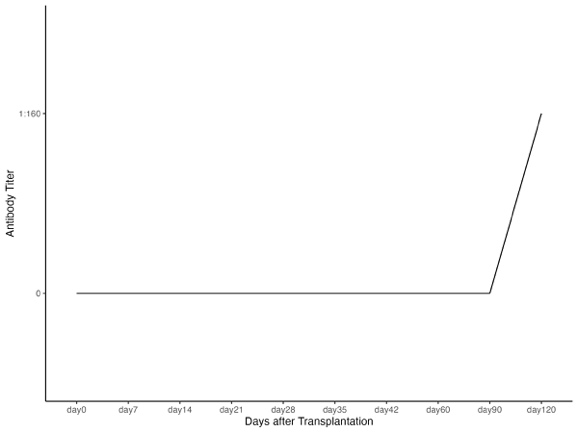 |
| 35 | STP | 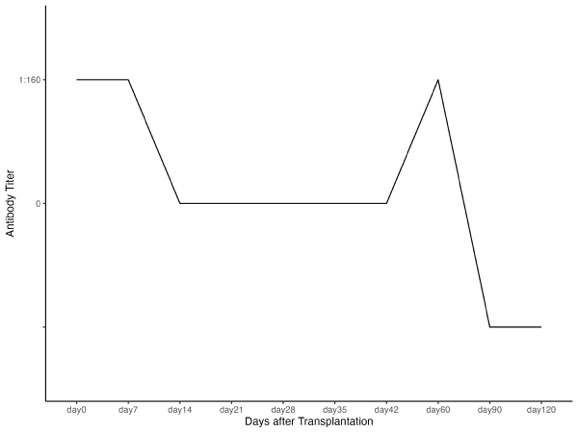 |
| 36 | CCP | 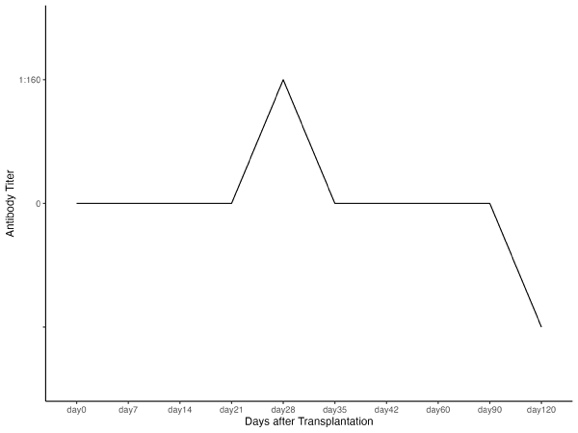 |
| 37 | CCP | 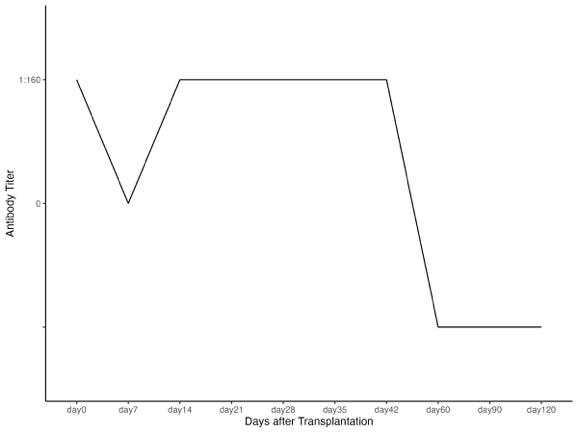 |
| 38 | CCP | 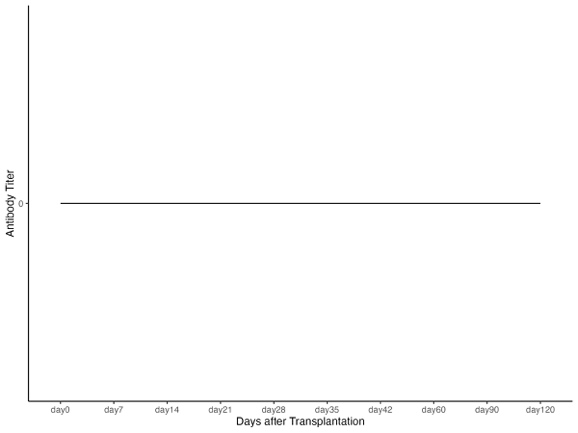 |
| 39 | STP | 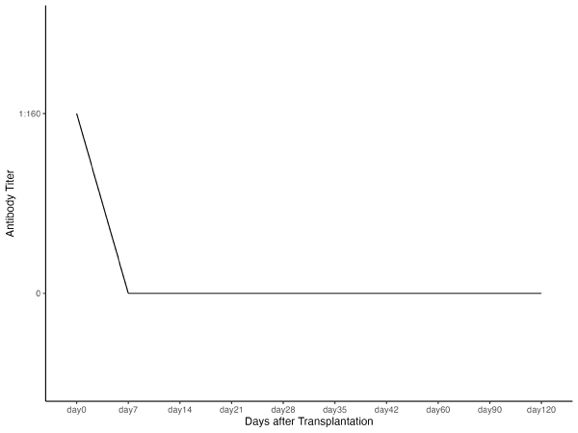 |
| 40 | CCP | 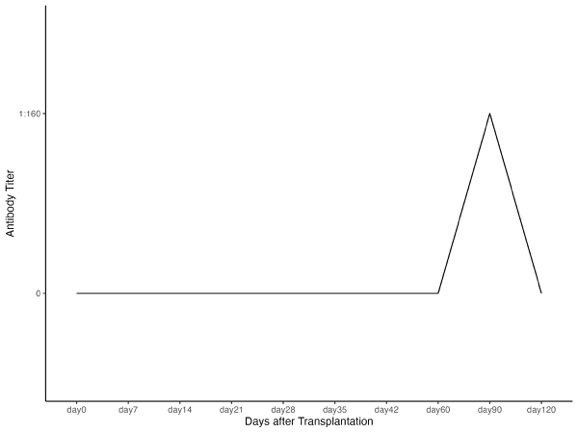 |
| 41 | CCP | 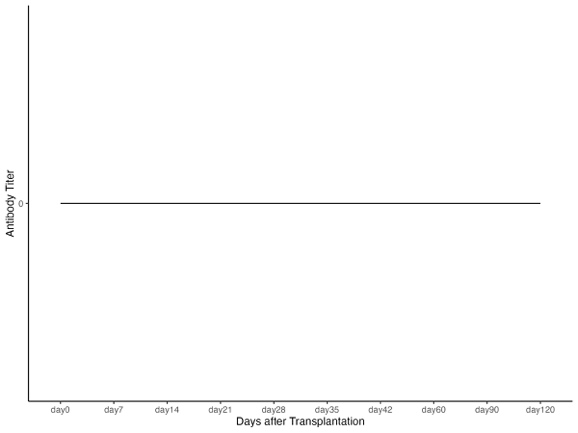 |
| 42 | STP | 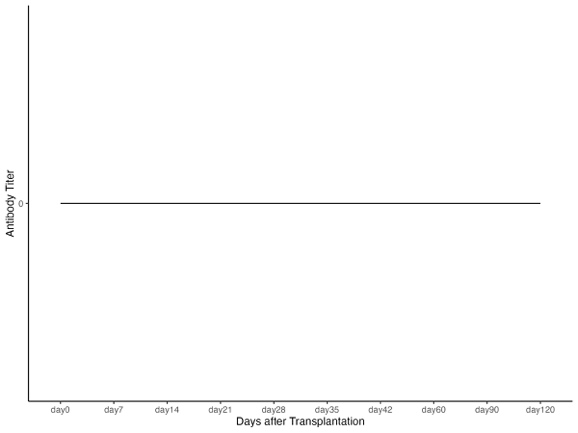 |
| 43 | STP | 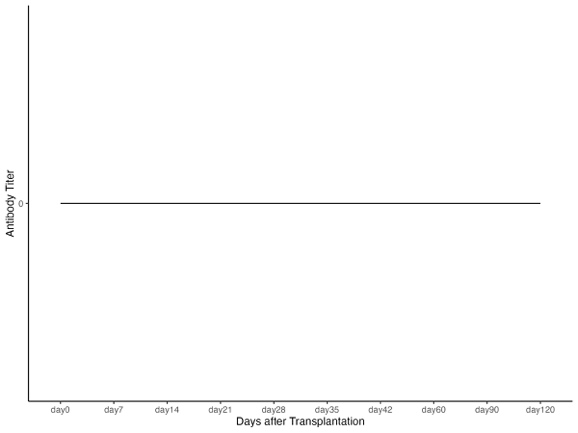 |
| 44 | STP | 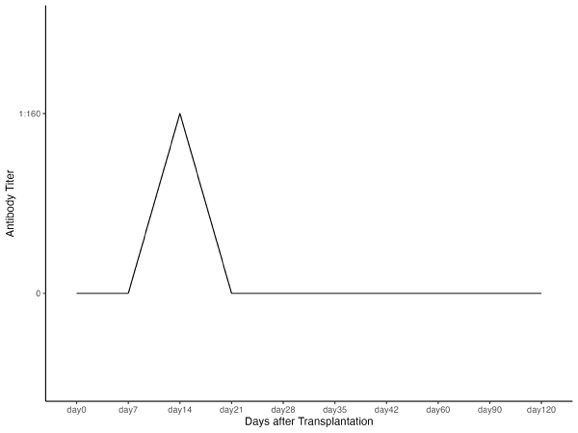 |
| 45 | CCP | 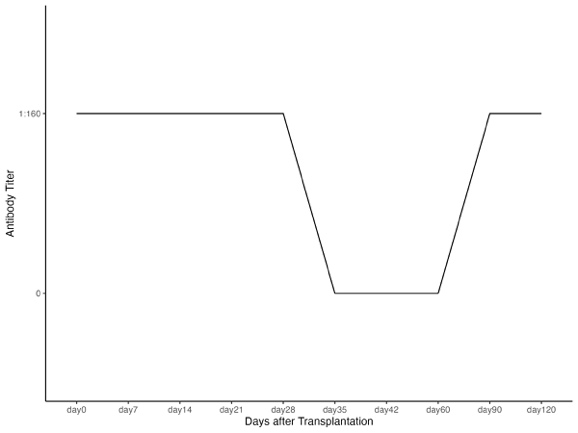 |
| 46 | CCP | 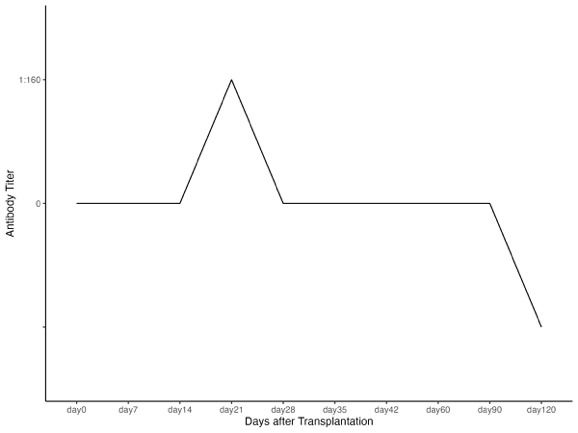 |
| 47 | STP | 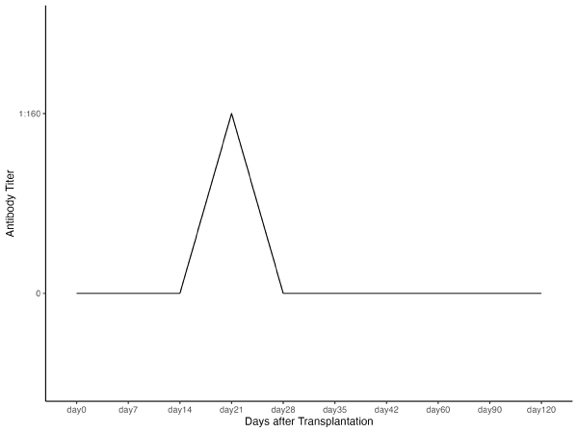 |
| 48 | CCP | 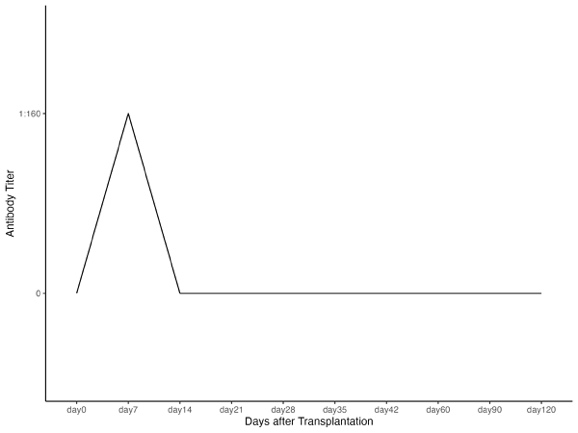 |
| 49 | CCP | 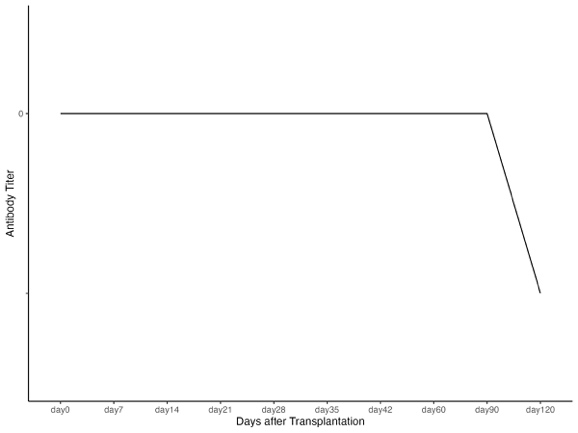 |
| 50 | CCP | 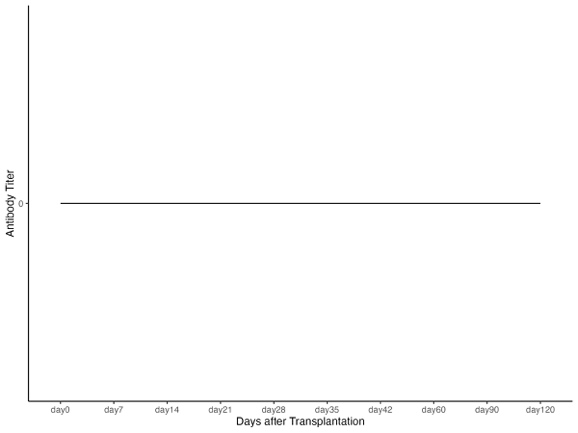 |
| 51 | STP | 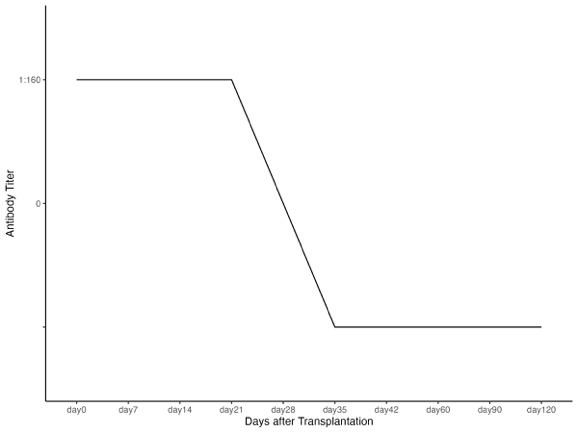 |
| 52 | STP | 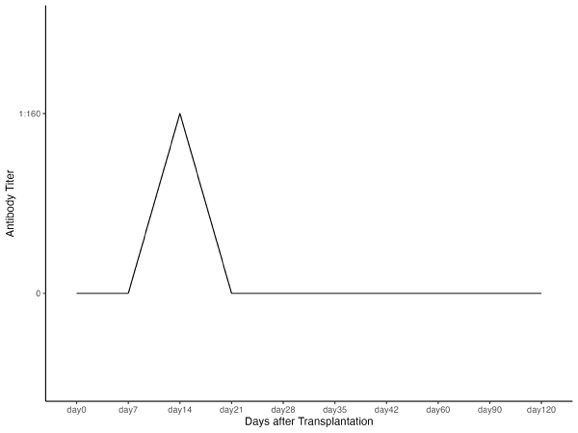 |
| 53 | STP | 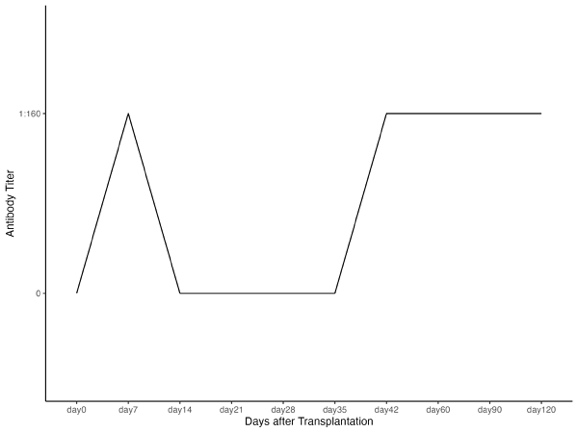 |
| 54 | STP | 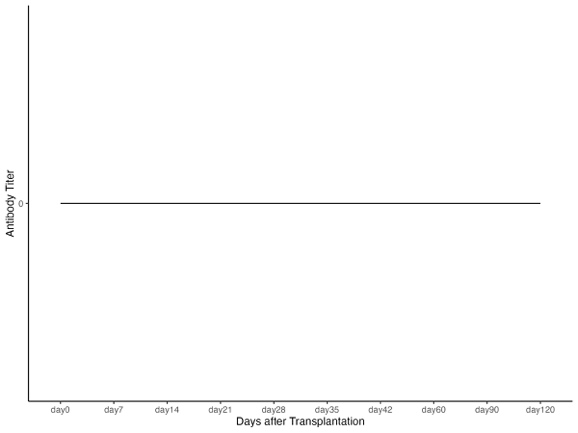 |
| 55 | CCP | 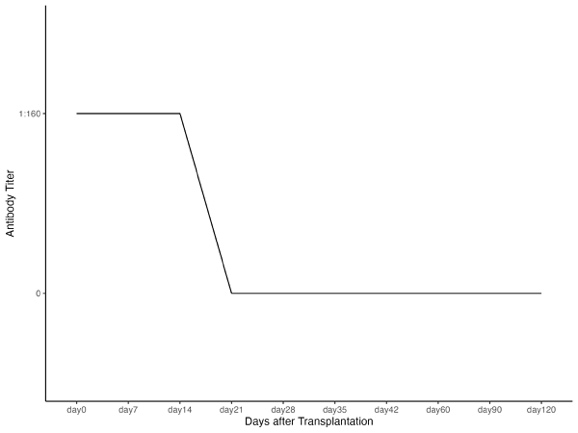 |
| 56 | CCP | 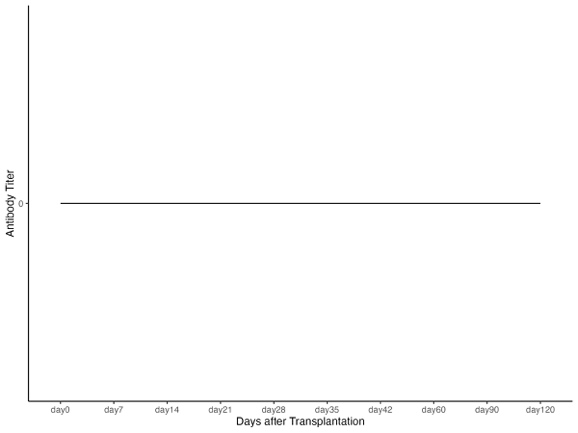 |
| 57 | CCP | 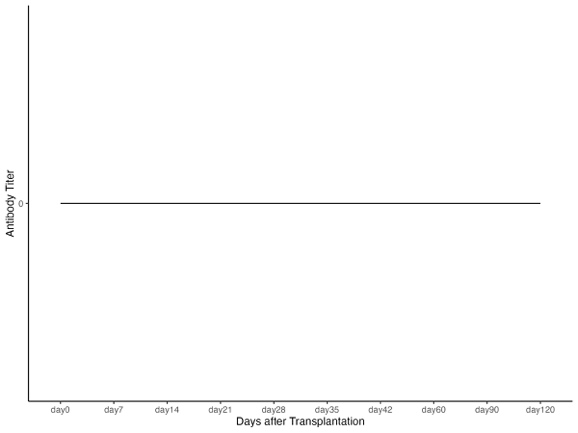 |
| 58 | STP | 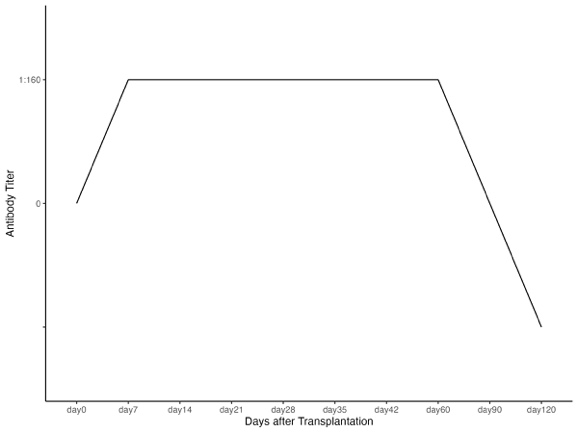 |
| 59 | STP | 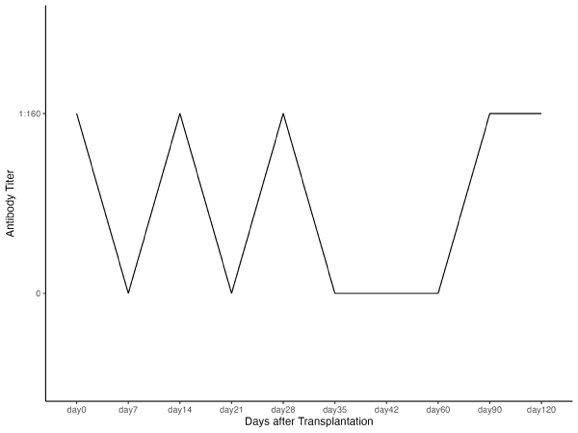 |
| 60 | CCP | 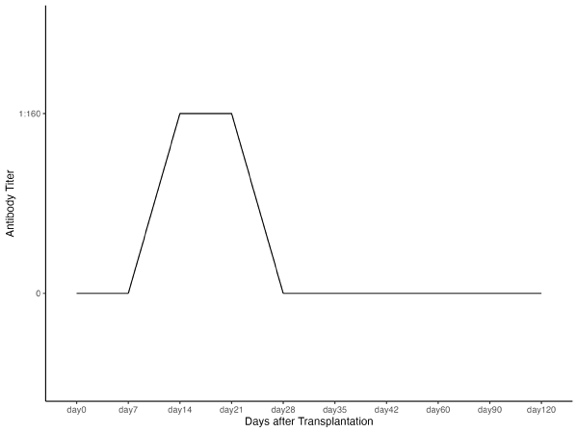 |
| 61 | STP | 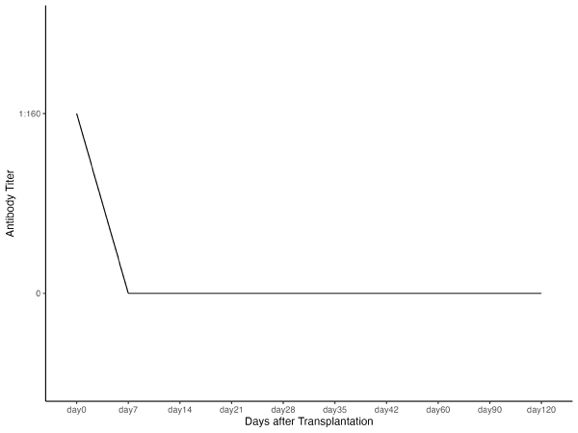 |
| 62 | STP | 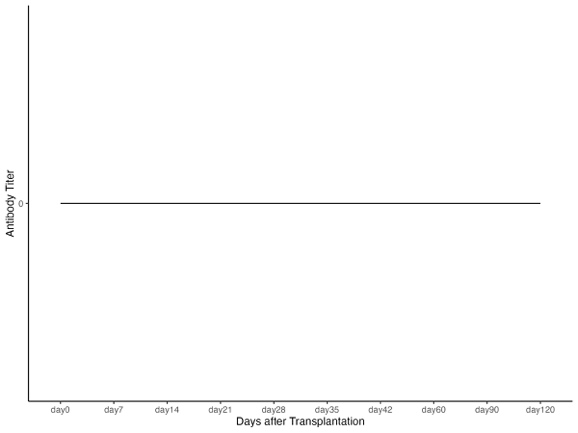 |
| 63 | STP | 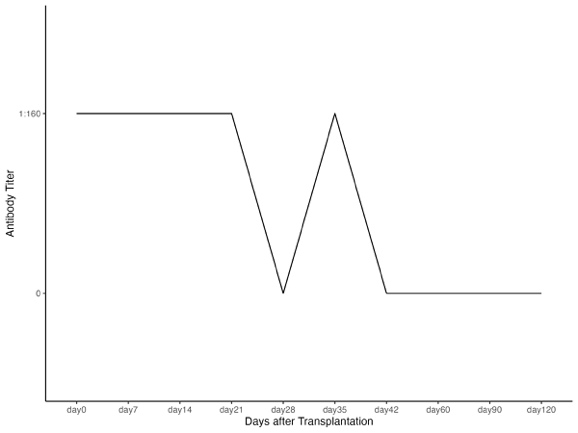 |
| 64 | CCP | 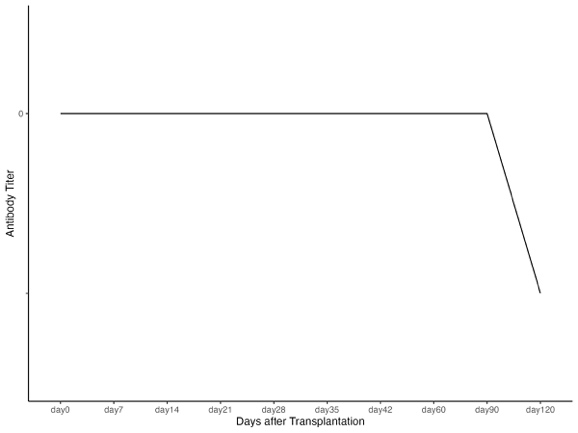 |
| 65 | STP | 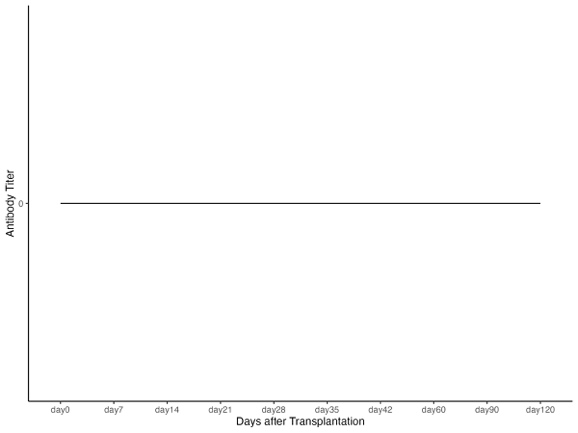 |
| 66 | CCP | 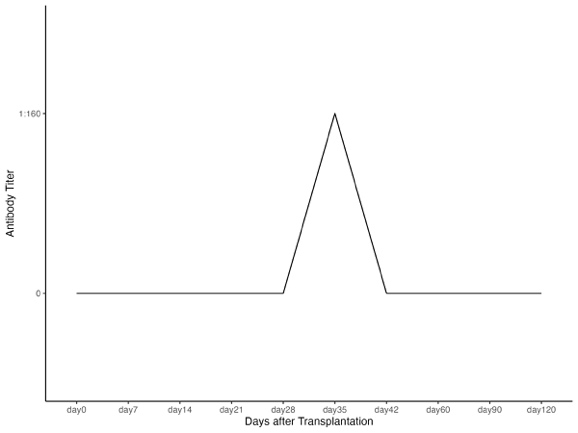 |
| 67 | CCP | 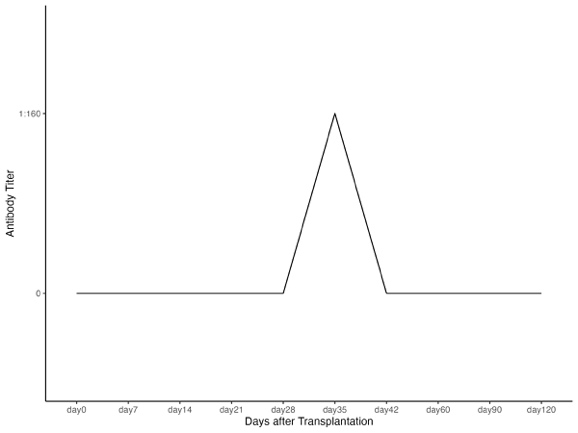 |
| 68 | CCP | 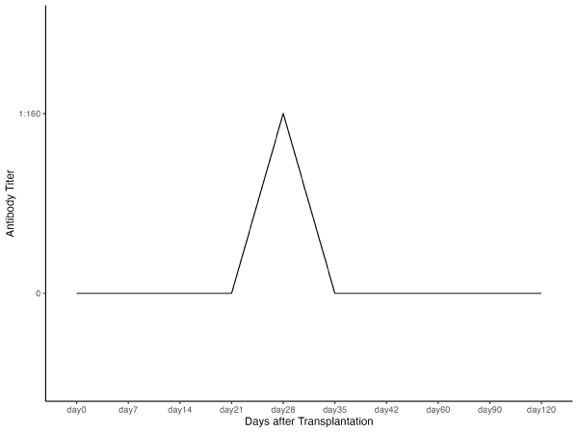 |
| 69 | STP | 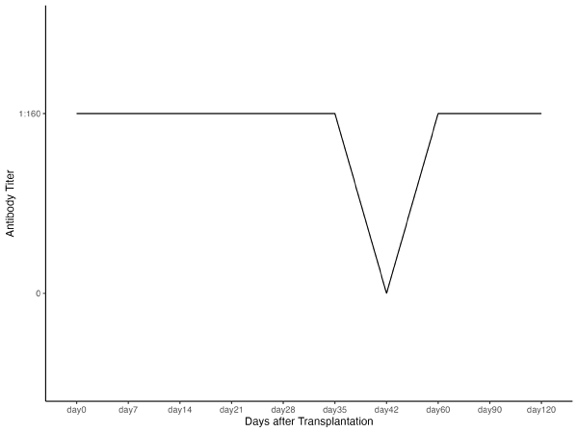 |
| 70 | CCP | 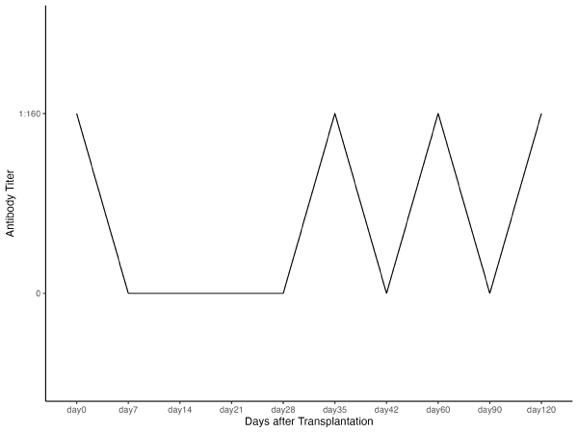 |
| 71 | CCP | 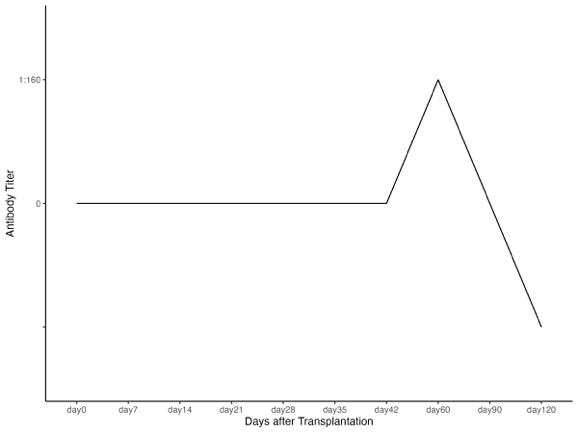 |
| 72 | CCP | 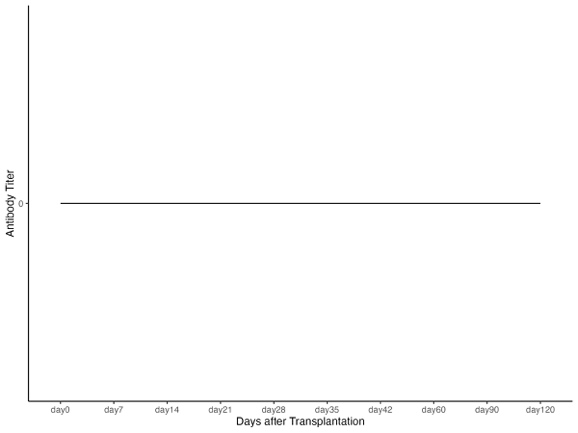 |

# Supplementary Figure 1


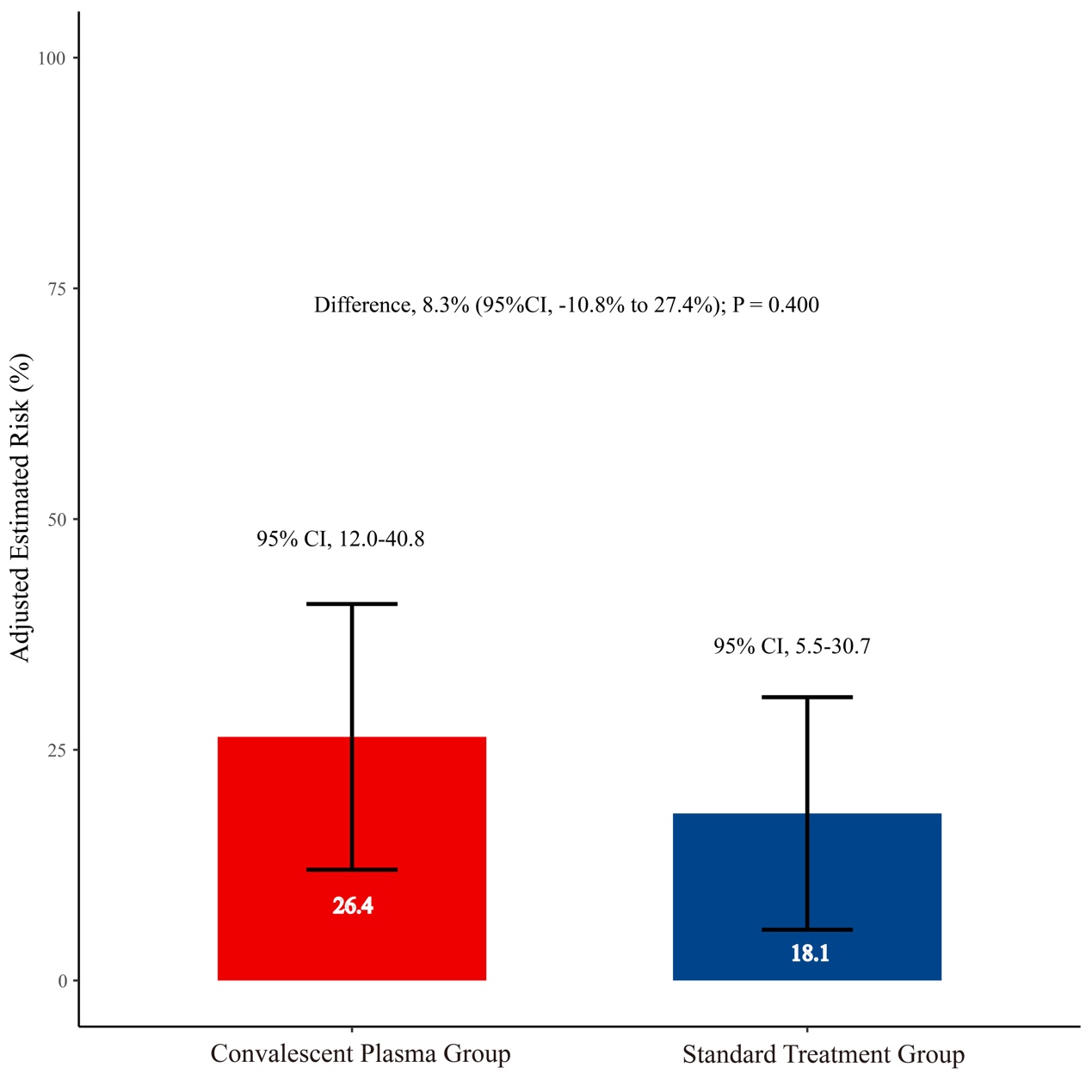


Supplement Fig. 1. The estimated adjusted risk of COVID-19 infection at 120 days.

Adjusting for age, sex, and HCT-CI scores stratification, the estimated adjusted risk of COVID-19 infection at 120 days was assessed.

# Supplementary Figure 2


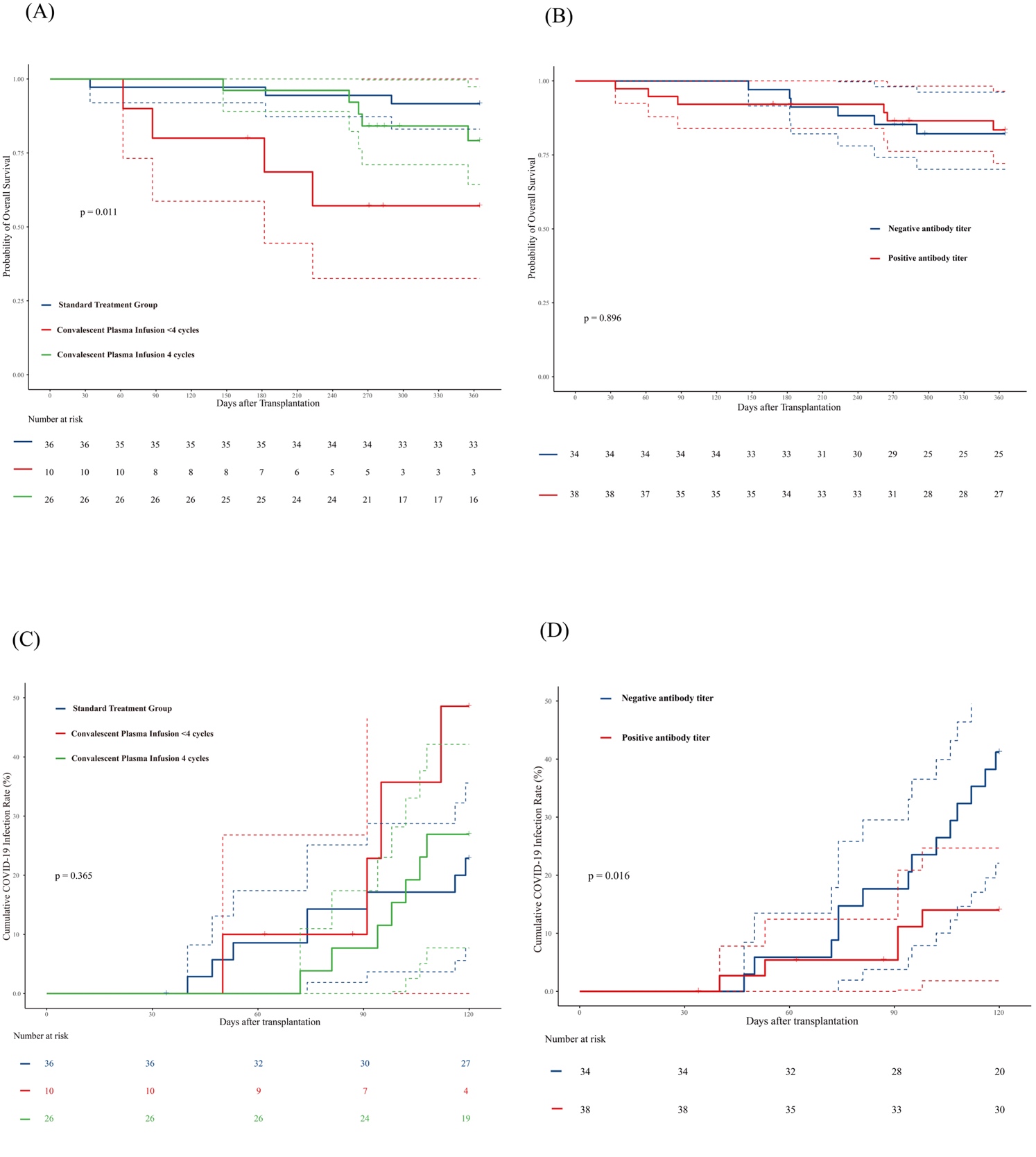


Supplementary Figure 2. Overall survival (OS) and COVID-19 infection, stratified by the number of CCP infusions and antibody titer

(A) One-year OS after HSCT by number of CCP infusions

(B) One-year OS after HSCT by antibody titer

(C) Cumulative incidence of COVID-19 infection by number of CCP infusions

(D) Cumulative incidence of COVID-19 infection by antibody titer

# Supplementary Figure 3


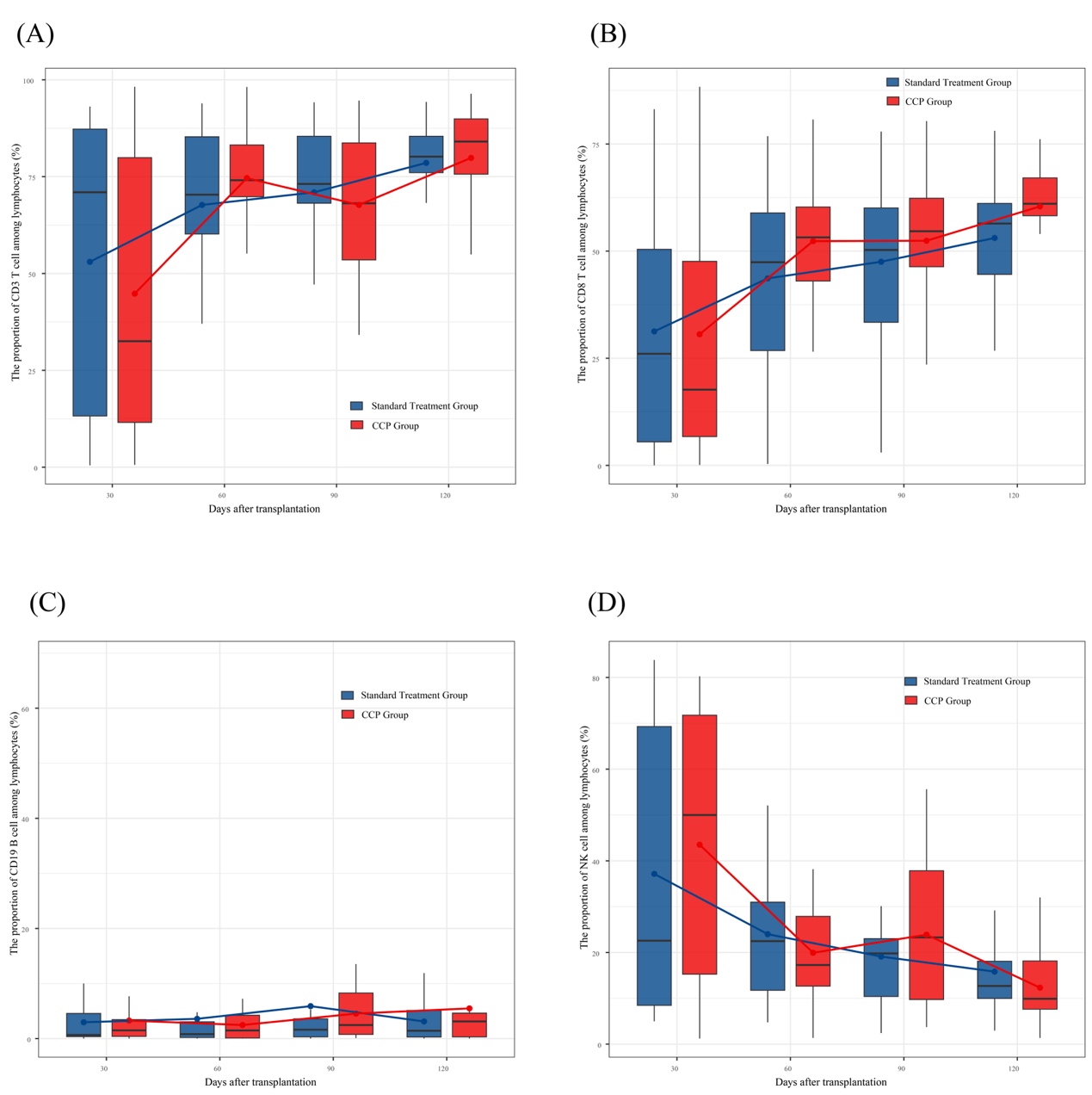


Supplement Fig. 3. Post-transplantation immune reconstitution

A. The distribution of peripheral blood CD3+ T-cell frequency (%); B. The distribution of peripheral blood CD8+ T-cell frequency (%); C. The distribution of peripheral blood CD19 B cell frequency (%); D. The distribution of peripheral blood NK cell frequency (%)

Boxes represent the first and third quartiles; lines inside boxes represent median values, with whiskers extending to 1.5 times the interquartile range (IQR) from the quartiles; circles inside boxes represent mean values.

# Supplementary Figure 4


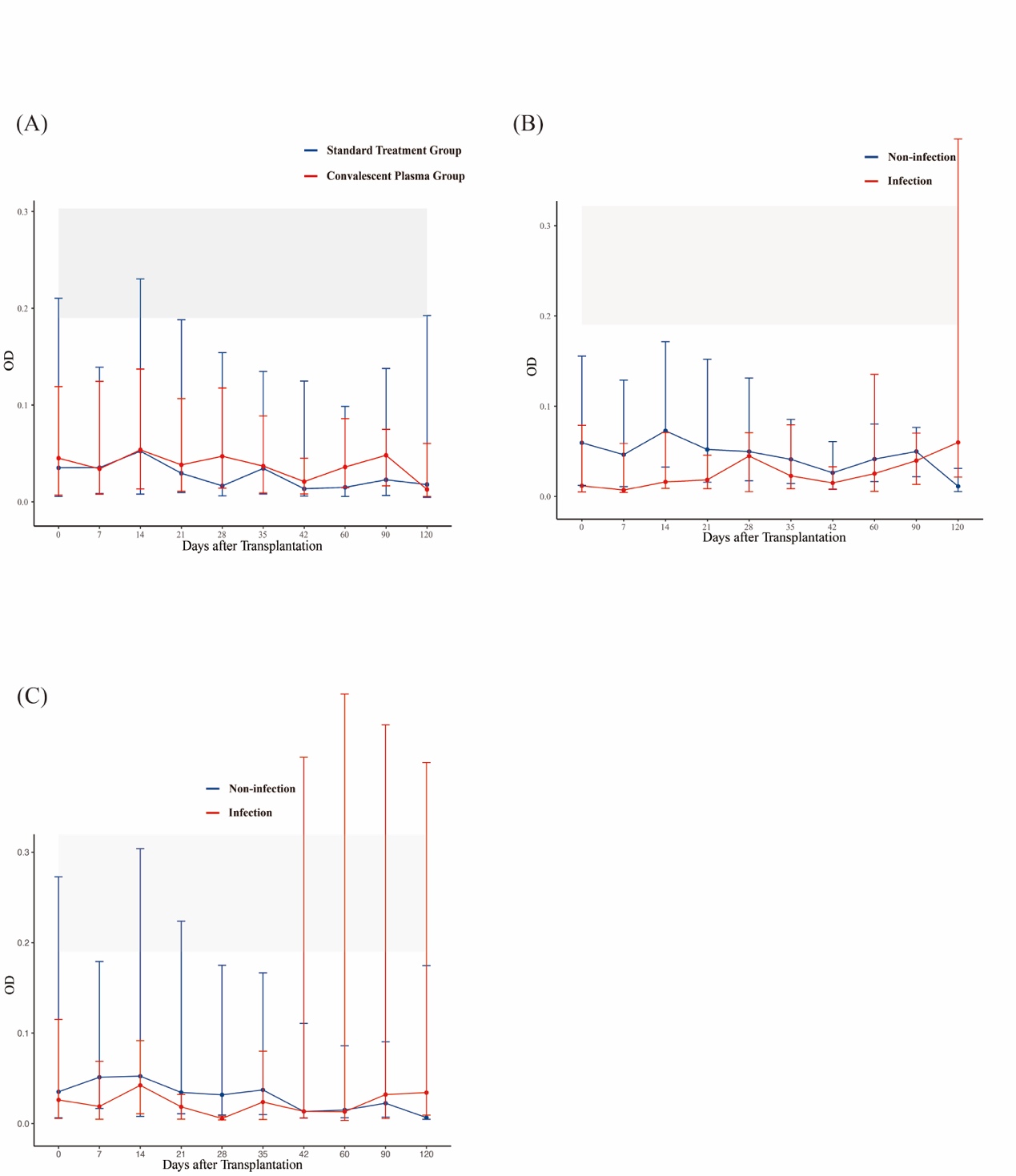


Supplement Fig. 4. Post-transplantation optical density (OD) variations in ITT populations

(A) shows the CCP and Standard treatment group, panel (B) depicts the infection (n = 11) and non-infection (n = 25) patients in CCP group, and panel (C) shows the infection (n = 8) and non-infection (n = 28) patients in the standard treatment group. Data are presented as medians with interquartile ranges. The gray zone represents the positive range with a cutoff value of 0.19.

# Supplementary Figure 5


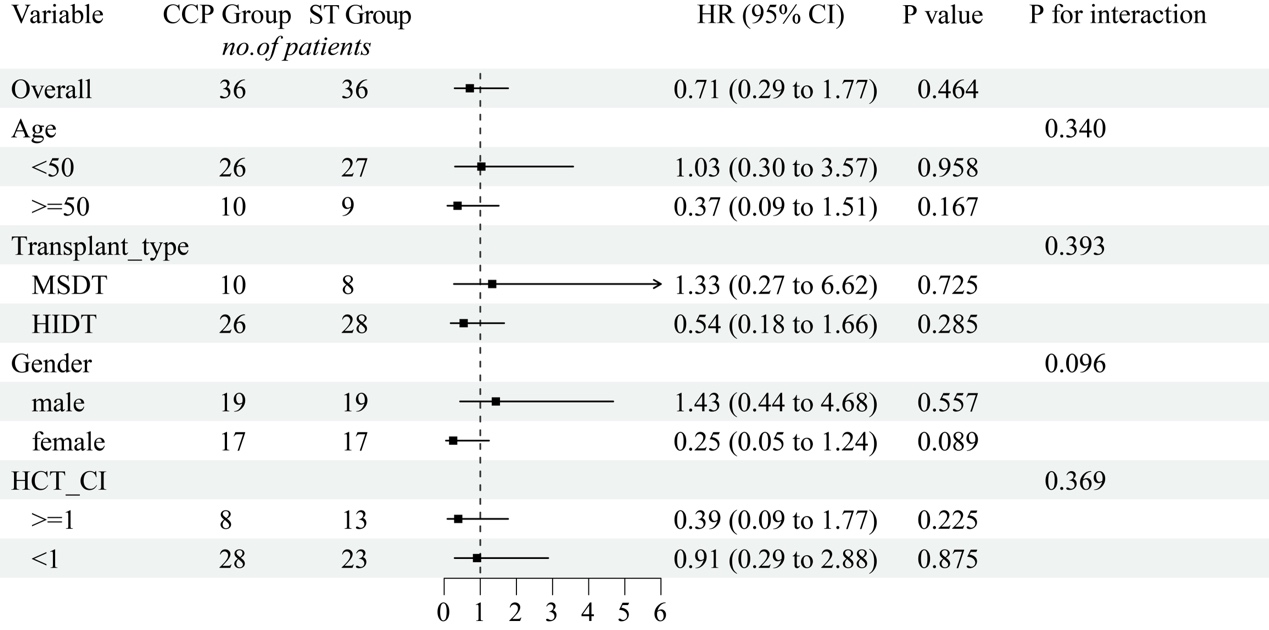


Supplement Fig. 5. Subgroup analysis in ITT populations

# Detailed Methodology for Antibody Testing

## New Coronavirus (2019-nCoV) IgG Antibody Detection Kit (Enzyme-Linked Immunosorbent Assay)

**1. Product Name**

Generic Name: New Coronavirus (2019-nCoV) IgG Antibody Detection Kit (Enzyme-Linked Immunosorbent Assay)

**2. Packaging Specifications**

48 tests/box, 96 tests/box

**3. Intended Use**

This kit is intended for the qualitative detection of 2019-nCoV IgG antibodies in human serum or plasma samples. Coronaviruses, which belong to the family Coronaviridae and the genus Coronavirus, are enveloped, positive-sense, single-stranded RNA viruses with a diameter of approximately 80-120nm. Their genetic material is the largest among all RNA viruses and they infect humans, rodents, pigs, cats, dogs, and birds. A variant of the coronavirus is the pathogen causing atypical pneumonia and is an RNA virus. Coronaviruses were first isolated from chickens in 1937, with virus particles ranging from 60-200nm in diameter, with an average diameter of 100nm, and are spherical or oval in shape, exhibiting pleomorphism. The virus has an envelope with protruding spikes, and the entire virus resembles a solar corona, with distinct differences in the spikes among different coronaviruses. Tubular inclusion bodies can sometimes be seen inside coronavirus-infected cells. 2019-nCoV-IgM antibodies appear earlier than 2019-nCoV-IgG antibodies, and after the acute phase, 2019-nCoV-IgM antibodies decline rapidly, while 2019-nCoV-IgG antibodies may persist for a long time. This kit is suitable for the auxiliary diagnosis of clinical novel coronavirus infections and should not be used as the sole basis for clinical diagnosis.

**4. Principle of the Test**

The kit uses the indirect enzyme-linked immunosorbent assay (ELISA) principle. The microwell strip is pre-coated with 2019-nCoV antigen, which can bind with 2019-nCoV antibodies in the sample. After the addition of enzyme-labeled reagent and incubation, the presence of 2019-nCoV-IgG antibodies in the sample will form a "coated antigen-antibody-enzyme-labeled secondary antibody" complex. The HRP on the complex catalyzes the color development agent to produce a blue product, which turns yellow after the termination reaction. If there are no 2019-nCoV-IgG antibodies in the sample, no color development occurs.

**5. Main Components**

1. Enzyme-labeled plate: 4 strips with 12 wells each for the 48T kit, and 8 strips with 12 wells each or 12 strips with 8 wells each for the 96T kit.

2. Positive control: 0.5 mL per vial for the 48T kit, 0.5 mL per vial for the 96T kit.

3. Negative control: 0.5 mL per vial for the 48T kit, 0.5 mL per vial for the 96T kit.

4. Sample diluent: 6 mL per bottle for the 48T kit, 12 mL per bottle for the 96T kit.

5. Enzyme-labeled reagent: 6 mL per bottle for the 48T kit, 12 mL per bottle for the 96T kit.

6. Concentrated wash solution: 20 times 30 mL per bottle for the 48T kit, 50 mL per bottle for the 96T kit.

7. Substrate solution A: 3 mL per bottle for the 48T kit, 6 mL per bottle for the 96T kit.

8. Substrate solution B: 3 mL per bottle for the 48T kit, 6 mL per bottle for the 96T kit.

9. Stop solution: 3 mL per bottle for the 48T kit, 6 mL per bottle for the 96T kit.

10. Self-sealing bag: 1 piece for the 48T kit, 1 piece for the 96T kit.

11. Sealing film: 2 pieces for the 48T kit, 2 pieces for the 96T kit.

Composition of each component:

Enzyme-labeled plate: Coated with 2019-nCoV antigen. Enzyme-labeled reagent: Contains horseradish peroxidase (HRP) labeled anti-human IgG antibody. Sample diluent: Contains protein buffer solution. Positive control: Contains 2019-nCoV-IgG positive material. Negative control: Contains protein buffer solution. Concentrated wash solution: Contains a surfactant with a concentration of not less than 2.5%. Substrate solution A: Contains hydrogen peroxide with a concentration of not less than 0.3g/L. Substrate solution B: Contains TMB with a concentration of not less than 0.2g/L. Stop solution: Contains sulfuric acid with a concentration not higher than 2M.

Note: The sealing film is not reusable, different batches of enzyme-labeled plates, enzyme-labeled reagents, and positive/negative controls should not be mixed, and should not be used with reagents from other manufacturers.

**6. Storage Conditions and Shelf Life**

The kit should be stored at 2~8°C with a shelf life of 12 months. Before use, allow the kit to equilibrate to room temperature (about 30 minutes). Unused microwell strips must be sealed with a desiccant in a self-sealing bag and stored at 2~8°C. Before the experiment, gently mix the liquid reagents, and immediately reseal and store them at 2~8°C after use. The production date and expiration date can be found on the label.

**7. Applicable Equipment**

Pipettes, incubators, plate washers, and ELISA readers with a wavelength of 450nm.

**8. Sample Requirements**

1. Sample Type: This kit is used with human serum or plasma samples. Samples containing anticoagulants such as EDTA, citrate, or heparin can be used for this test.

2. Interference Factors: Common interfering factors such as hemolysis (hemoglobin content not exceeding 400mg/L), hyperlipidemia (triglyceride content not exceeding 170 mmol/L), jaundice (bilirubin content not exceeding 1.71mmol/L), and positive rheumatoid factor will not cause false positives with this kit. Samples containing suspended fibrin or aggregates cannot be tested.

3. Sample Storage: The samples should be free from microorganisms. Aseptically separated samples can be stored at 2–8°C for 1 week. For long-term storage, samples should be frozen below -15°C. Samples should not be subjected to more than 3 freeze-thaw cycles.

4. Sample Equilibration: Before use, please allow the samples to equilibrate at room temperature for more than 30 minutes. Thawed samples should be mixed well before the experiment.

**9. Test Procedure**

1. Dilution: Dilute the concentrated wash solution with distilled water or deionized water at a ratio of 20:1.

2. Numbering: Number the samples corresponding to the microwell plate in sequence, with 3 negative control wells, 2 positive control wells, and 1 blank control well for each plate. (If dual-wavelength detection is used, the blank control well can be omitted.)

3. Dilution: Add 100μL of sample diluent to each well, except for the blank and negative/positive control wells.

4. Sample addition: Add 10μL of the test sample to the corresponding wells, and 100μL of negative/positive control to their respective wells, then gently mix.

5. Incubation: Seal the plate with a sealing film and incubate at 37°C for 30 minutes.

6. Washing: Carefully remove the sealing film and wash the plate with a plate washer for 5 times, making sure to drain as much as possible during the last wash.

7. Enzyme addition: Add 100μL of enzyme-labeled reagent to each well, except for the blank well.

8. Incubation: Same as step 5.

9. Washing: Same as step 6.

10. Color development: Add 50μL of substrate solution A and B to each well, gently mix, and incubate at 37°C in the dark for 15 minutes.

11. Reading: Add 50μL of stop solution to each well, gently mix, and read the results within 10 minutes. Set the ELISA reader wavelength to 450nm (dual-wavelength 450nm/600~650nm detection is recommended), adjust the zero point with the blank well, and then measure the A values of each well.

**10. Positive Value**

1. Cutoff value calculation: Cutoff value = 0.16 + mean A value of the negative control wells (if the negative control well is below 0.03, calculate it as 0.03).

2. The A value of the negative control well should be ≤0.10, and the A value of the positive control well should be ≥0.19; otherwise, the test is invalid.

3. If one negative control well has an A value greater than 0.1, it should be discarded. If two or more negative control wells have A values greater than 0.1, the experiment should be repeated.

**11. Interpretation of Test Results**

1. Positive determination: If the sample A value is ≥ the cutoff value (CUTOFF), it is considered 2019-nCoV-IgG antibody positive.

2. Negative determination: If the sample A value is < the cutoff value (CUTOFF), it is considered 2019-nCoV-IgG antibody negative.

**12. Limitations of the Test Method**

1. The results of this kit must be analyzed in conjunction with clinical information and cannot be used as the sole basis for clinical diagnosis.

2. A negative test result does not rule out the possibility of 2019-nCoV infection. During the early stages of 2019-nCoV infection, in immunocompromised patients or those receiving immunosuppressive therapy, the level of serological antibody content may be limited, and the limitations of the ELISA reaction principle may also lead to such results. It is recommended that patients be retested within 7-14 days, and the specimens collected previously should be tested in parallel to confirm whether there is a seroconversion or a significant increase in titer.

3. This kit cannot be used as a quantitative reagent.

4. This kit is only intended for the detection of human serum or plasma samples.

**13. Product Performance Indicators**

The kit meets national standards when tested with the national reference material. When tested with the enterprise reference material, it meets the following criteria: the positive reference materials P1~P2 have a compliance rate of 2/2; the negative reference materials N1~N10 have a compliance rate of 10/10. For the detection limit reference materials (S1~S5), S1~S3 should test positive, S4 can be positive or negative, and S5 should test negative. The coefficient of variation (CV%) should not be higher than 15%.

**14. Precautions**

1. This product is for in vitro diagnostic use only. The operation should be carried out strictly according to the instructions. Users must be professionally trained. Before conducting the experiment, wear masks, gloves, and lab coats.

2. The sealing film should not be reused. Enzyme-labeled plates, enzyme-labeled reagents, and positive/negative controls from different batches should not be mixed, and should not be used with reagents from other manufacturers.

3. Avoid operating in environments with volatile substances and hypochlorous acid disinfectants (such as 84 disinfectant).

4. Before use, allow the reagents to equilibrate to room temperature (for 30 minutes), gently mix the liquid reagents before the experiment, and immediately reseal and store them at 2-8°C after use. Unused microwell strips should be sealed with a desiccant in a self-sealing bag and stored at 2-8°C. Do not use expired reagents and avoid microbial contamination.

5. Positive and negative controls are already diluted and ready to use directly.

6. When adding liquids, a pipette must be used, and the accuracy of the pipette should be checked regularly. The pipette tip and the pipette slot should be changed when adding different samples or different reagent components to prevent cross-contamination.

7. When washing, each well must be filled with wash solution to ensure that free enzymes in the well are washed away. The plate washer should be set for a soaking time of 30-60 seconds. After washing, proceed immediately to the next step without drying the enzyme-labeled plate. Avoid long interruptions in the experimental steps to ensure uniform experimental conditions in each well.

8. The result judgment must be based on the readings from the ELISA reader. When reading the results, dry the bottom of the microwell plate, and ensure there are no bubbles in the wells. Do not touch the bottom of the wells, as fingerprints or scratches may affect the readings.

9. The positive and negative controls in this product have been inactivated, and are negative for HBsAg, anti-HIV, anti-HCV, and anti-TP. However, they should still be handled as potentially infectious samples. All samples, waste liquids, and waste should be treated as infectious substances. The stop solution is sulfuric acid, so handle with care. If you accidentally come into contact with irritating liquids or infectious substances, rinse thoroughly with plenty of water and seek medical attention if necessary.

10. During color development, add the substrate solution A before adding solution B to avoid low color development.

11. Key equipment used in the experiment should be regularly calibrated and maintained.

**15. Basic Information**

Registration Holder/Manufacturer: Beijing Wantai Biological Pharmacy Enterprise Co., Ltd.

Address: No. 31 Science Park Road, Changping District, Beijing

Contact: 010-59528888

After-Sales Service Unit: Beijing Wantai Biological Pharmacy Enterprise Co., Ltd.

Service Contact: 400-650-5969

Production Address: No. 31 Science Park Road, Changping District, Beijing

Production License Number: Beijing Food and Drug Administration Medical Device Production Permit No. 20040097

Remark: the primary version of this Antibody Detection Kit protocol was in Chinese. We have translated it into English
