# Supplementary material for: Convalescent plasma therapy for COVID-19 prophylaxis in adults early post-hematopoietic stem cell transplantation: one-year outcomes from a randomized controlled trial
Source: Front Immunol. 2025 Nov 28;16:1626775. doi: 10.3389/fimmu.2025.1626775 (PMC12698628; doi:10.3389/fimmu.2025.1626775)
Supplement: Supplementary file 2 [file Table2.docx]

Supplementary Appendix1

**Original Clinical Trial Protocol (English Translation)**

**Protocol Title:** Prophylactic Use of COVID-19 Convalescent Plasma in Adult Patients Early After Hematopoietic Stem Cell Transplantation (CCP-COVID-19-HSCT-2023, Version 6.0)

**Approved:** January 7, 2023

**Institution:** Tianjin Institute of Hematology, Chinese Academy of Medical Sciences & Peking Union Medical College

**Principal Investigator:** Dr. Yigeng Cao

**I. Study Summary**

**Study Title:**

Prophylactic Use of COVID-19 Convalescent Plasma in Adult Patients Early After Hematopoietic Stem Cell Transplantation: A Randomized Controlled Trial.

**Protocol Number:** CCP-COVID-19-HSCT-2023-V6.0

**Version and Date:** Version 6.0, January 7, 2023

**Study Phase:** Investigator-initiated clinical study (non-commercial, single-center, randomized controlled trial).

**Sponsor Institution:** Department of Hematopoietic Stem Cell Transplantation, Tianjin Institute of Hematology, CAMS & PUMC.

**Principal Investigator:** Dr. Yigeng Cao, Deputy Chief Physician.

**Study Objective:**

To evaluate whether prophylactic infusion of high-titer COVID-19 convalescent plasma (CCP) can reduce the incidence of COVID-19 infection during the early post-transplant period in adult HSCT recipients, compared with standard supportive care alone.

**Study Population:**

Adult patients (aged ≥18 years) within 100 days after allogeneic HSCT, without active SARS-CoV-2 infection at enrollment.

**Study Design:**

A prospective, single-center, open-label, randomized controlled trial.

Eligible participants are randomized 1:1 to receive either:

1. CCP group: Four planned infusions of high-titer CCP (200 mL each) at day +14, +28, ~2 months, and ~3 months post-transplant.
2. Control group: Standard supportive care without CCP.

All participants are followed for one year after transplantation.

**Primary Endpoint:**

Incidence of laboratory-confirmed COVID-19 infection within one year after HSCT.

**Secondary Endpoints:**

1. Incidence and severity of COVID-19 (mild/moderate/severe).
2. Overall survival (OS) at one year.
3. Time to first SARS-CoV-2 infection.
4. Incidence of adverse events related to plasma infusion.
5. SARS-CoV-2 antibody titers and their kinetics over time.

**Sample Size:**

72 participants (36 per group), calculated assuming infection rates of 80% in the control group and 50% in the CCP group, α = 0.05, β = 0.20, using a log-rank test.

**Study Duration:**

Total study period: 12 months after last enrollment.

Each participant: 12 months of post-transplant follow-up.

**Statistical Analysis:**

Both intention-to-treat (ITT) and per-protocol (PP) analyses will be performed. Kaplan–Meier survival curves will be generated, and group comparisons will use the log-rank test. Continuous variables will be compared using the t-test or Mann–Whitney U test; categorical variables using χ² or Fisher’s exact test.

**Safety Monitoring:**

Adverse events (AEs) and serious AEs (SAEs) will be graded according to CTCAE v5.0, summarized by severity, relationship to study treatment, and reported to the institutional ethics board.

**Ethical Considerations:**

The study is conducted in accordance with the Declaration of Helsinki (2013), GCP (ICH E6 R2), and Chinese regulatory guidelines. Written informed consent is obtained from all participants.


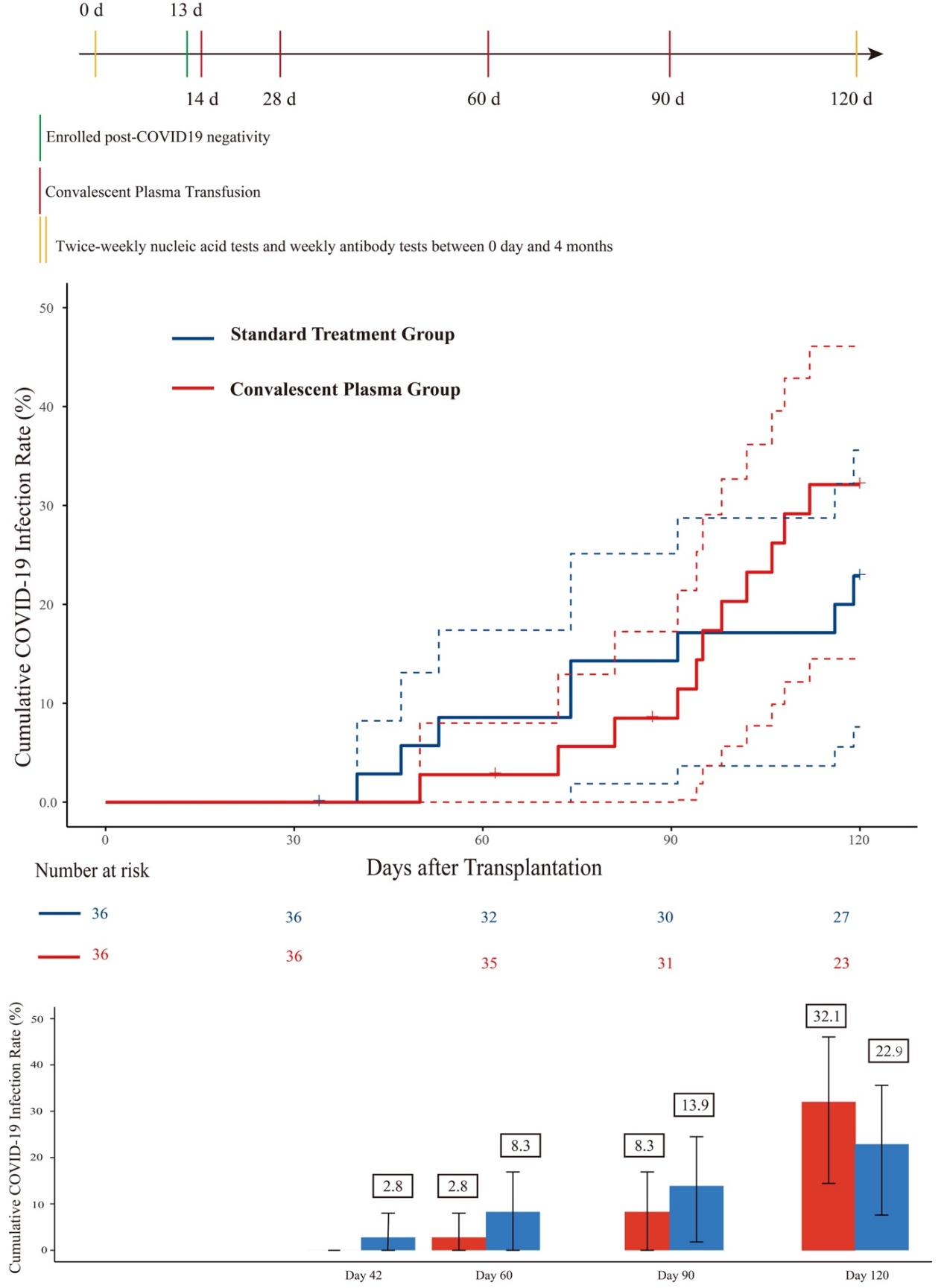


**Figure 1. Study schedule.**

**Timeline of convalescent plasma (CCP) infusions and virological/immunological monitoring after HSCT. CCP infusions were given on days +14, +28, +60, and +90. Twice-weekly nucleic acid and weekly antibody testing continued through day +120.**

**Ⅱ. Background and Rationale**

2.1 Background

Since its emergence in late 2019, coronavirus disease 2019 (COVID-19), caused by severe acute respiratory syndrome coronavirus 2 (SARS-CoV-2), has led to an unprecedented global pandemic. Despite the widespread implementation of vaccination programs, breakthrough infections and the emergence of new viral variants continue to pose serious threats to immunocompromised populations, particularly hematopoietic stem-cell transplantation (HSCT) recipients.

HSCT recipients represent one of the most vulnerable groups to COVID-19 due to profound and prolonged immune deficiency. Multiple studies have demonstrated that, in the early post-transplant period, patients experience severe lymphopenia, delayed T- and B-cell recovery, and diminished humoral and cellular responses to vaccination. Consequently, they remain at exceptionally high risk for prolonged viral shedding, severe infection, and death. The European Society for Blood and Marrow Transplantation (EBMT) and the American Society for Transplantation and Cellular Therapy (ASTCT) have both identified COVID-19 as a major post-transplant complication requiring preventive strategies beyond vaccination alone.

2.2 Rationale for Convalescent Plasma Therapy

COVID-19 convalescent plasma (CCP) contains high-titer polyclonal antibodies derived from individuals who have recovered from SARS-CoV-2 infection. These antibodies can provide passive immunity by neutralizing circulating virions, blocking viral entry into host cells, facilitating antibody-dependent cellular cytotoxicity (ADCC), and modulating inflammatory cytokine cascades.

Several studies have shown that CCP therapy is most effective when administered early in the disease course or to individuals lacking endogenous antibodies, such as immunocompromised patients and those with impaired humoral immunity.

In HSCT recipients, especially during the first 100 days post-transplant, endogenous antibody production is minimal due to delayed B-cell recovery and ongoing immunosuppressive therapy. This period corresponds to the highest incidence of viral infections and infectious mortality. The rationale for using CCP in this population is to bridge the “immune gap” between transplantation and vaccine-induced or natural antibody generation, thereby reducing early post-transplant infection risk.

2.3 Scientific Basis for Timing and Dosing

The CCP infusion schedule in this study—at day +14, day +28, approximately 2 months, and 3 months post-transplant—was designed to align with both immunologic recovery kinetics and clinical milestones in HSCT care:

1. Day +14: Represents the period of deepest lymphopenia and hypogammaglobulinemia, coinciding with protective-isolation discontinuation or discharge following engraftment. Passive antibody transfer at this stage provides coverage when vaccine response is absent.
2. Day +28: The half-life of IgG (~21 days) suggests waning antibody levels after the first infusion; a second dose extends protection during ongoing immune insufficiency.
3. ~2 months: Around day +60 marks the immunologic “transition point,” when partial lymphocyte recovery occurs but humoral immunity remains immature.
4. ~3 months: Vaccine immunogenicity remains poor within 3–6 months post-HSCT; this dose bridges the gap until active immunity can be established.

This “bridge-immunity” strategy integrates pathophysiological and clinical-pathway considerations to maintain continuous protection throughout the most vulnerable early post-transplant phase.

2.4 Study Significance

Despite extensive clinical experience with convalescent plasma in severe or critical COVID-19 cases, data on its prophylactic use—particularly among HSCT recipients—remain extremely limited. Given the delayed immune reconstitution and poor vaccine responses in this population, early passive immunization represents a rational, low-toxicity approach to reduce infection risk and mortality.

This trial aims to provide high-quality prospective evidence regarding the feasibility, safety, and efficacy of prophylactic CCP administration in adult HSCT recipients, filling an important knowledge gap in post-transplant infectious prophylaxis.

**Ⅲ. Study Objectives and Endpoints**

3.1 Primary Objective

To evaluate whether prophylactic infusion of high-titer COVID-19 convalescent plasma (CCP) reduces the incidence of laboratory-confirmed SARS-CoV-2 infection within one year after allogeneic hematopoietic stem-cell transplantation (HSCT), compared with standard supportive care alone.

3.2 Secondary Objectives

1. To assess the severity and clinical course of COVID-19 infection (mild, moderate, severe, or critical) in CCP versus control groups.

2. To evaluate overall survival (OS) at one year post-transplant.

3. To determine the time to first SARS-CoV-2 infection after transplantation.

4. To assess the kinetics and durability of anti–SARS-CoV-2 antibody levels following CCP infusion.

5. To evaluate the safety and tolerability of CCP administration by recording infusion-related and systemic adverse events.

6. To explore correlations between antibody titers and clinical outcomes.

3.3 Primary Endpoint

Incidence of laboratory-confirmed SARS-CoV-2 infection within 12 months after HSCT.

3.4 Secondary Endpoints

1. COVID-19 severity grading according to WHO Clinical Progression Scale (scores 1–10).

2. Overall survival rate at 12 months post-transplant.

3. Time to first COVID-19 diagnosis (days from transplantation to first positive test).

4. Quantitative or semiquantitative SARS-CoV-2 antibody titers measured at predefined intervals (baseline, pre-infusion, and 14, 28, 60, 90, 180, and 365 days post-transplant).

5. Number and proportion of adverse events (AEs) and serious adverse events (SAEs) related to plasma infusion, graded according to CTCAE v5.0.

6. Association between CCP antibody titer and risk reduction for COVID-19 infection or severe disease.

3.5 Exploratory Endpoints

Correlation of CCP antibody titers (or binding absorbance values) with humoral reconstitution kinetics (IgG, IgM, IgA recovery).

Influence of donor type (matched sibling, haploidentical, or unrelated donor) and conditioning regimen on infection risk and CCP efficacy.

Subgroup analyses by patient age, gender, and comorbidities (e.g., diabetes, cardiovascular disease).

**Ⅳ. Study Design and Methodology**

4.1 Study Design

This is a prospective, single-center, open-label, randomized controlled trial evaluating the prophylactic efficacy and safety of COVID-19 convalescent plasma (CCP) in adult patients early after allogeneic hematopoietic stem-cell transplantation (HSCT).

Eligible participants are randomized in a 1:1 ratio to receive either:

Experimental group (CCP group): Standard supportive care plus four planned CCP infusions.

Control group: Standard supportive care without CCP.

The total study duration for each participant is 12 months after transplantation. Participants will be followed for clinical outcomes, infection status, and safety events throughout the observation period.

4.2 Randomization and Allocation Concealment

Randomization is performed using a computer-generated sequence prepared by an independent statistician who is not involved in patient care.

Allocation follows a simple randomization design with a 1:1 ratio between the CCP group and the control group. The randomization sequence is stored securely by the data manager and concealed until the time of patient enrollment.

Upon confirmation of eligibility and signed informed consent, the investigator obtains the next available randomization code from the sealed allocation list. This process ensures that treatment assignment remains concealed until enrollment is finalized.

4.3 Blinding

This is an open-label study; neither investigators nor participants are blinded to treatment allocation.

However, laboratory personnel responsible for SARS-CoV-2 testing and antibody titer quantification are blinded to treatment assignment to minimize analytical bias. Clinical investigators and patients were aware of group assignment solely for safety monitoring and transfusion logistics.

4.4 Intervention

4.4.1 Experimental Group (CCP Group)

Participants receive standard supportive care plus four infusions of high-titer COVID-19 convalescent plasma (CCP).

Dosage: 200 mL per infusion.

Infusion schedule:

1. Day +14 ± 2 days
2. Day +28 ± 2 days
3. ~2 months (±7 days)
4. ~3 months (±7 days)

Each CCP unit must meet the following criteria:

Collected from donors who have fully recovered from COVID-19 and are at least 14 days symptom-free.

Confirmed SARS-CoV-2 PCR negative at the time of donation.

Tested positive for anti–SARS-CoV-2 IgG with high antibody titer (S/CO ≥ 12) according to ELISA testing standards.

ABO compatible with the recipient.

Infusions are administered intravenously at a rate not exceeding 2 mL/kg/hour, with continuous monitoring for transfusion reactions.

4.4.2 Control Group

Participants in the control arm receive standard supportive care according to institutional post-HSCT guidelines, without plasma infusion.

4.5 Concomitant Medications and Standard Supportive Care

Both study arms follow the same post-HSCT standard of care, including:

Antimicrobial prophylaxis (bacterial, viral, and fungal).

GVHD prophylaxis (e.g., calcineurin inhibitors, methotrexate, or mycophenolate mofetil).

Blood component support (platelets, red blood cells) as clinically indicated.

Ursodeoxycholic acid (UDCA) is permitted as part of the institutional hepatoprotective regimen and is used equally in both groups; it is not considered a study intervention.

4.6 Safety Monitoring

All adverse events (AEs) and serious adverse events (SAEs) occurring during or after CCP infusion will be recorded.

Events are graded according to Common Terminology Criteria for Adverse Events (CTCAE) version 5.0, categorized by severity (Grade 1–5) and relationship to the study intervention (definite, probable, possible, unrelated).

All SAEs are reported within 24 hours to the principal investigator and reviewed by the institutional ethics committee.

An independent Data and Safety Monitoring Board (DSMB) oversees trial conduct and safety data.

4.7 Follow-Up and Assessments

| **Time Point** | **Clinical Assessments** | **Laboratory Assessments** |
| --- | --- | --- |
| Baseline (pre-transplant) | Demographics, medical history, HSCT details, donor type | SARS-CoV-2 antibody titer, PCR test |
| Day +14 | Vital signs, infusion tolerance | Antibody titer, CBC, liver/renal function |
| Day +28 | AE assessment, infection monitoring | Antibody titer, PCR test |
| 2 months | Clinical evaluation, GVHD status | Antibody titer, PCR test |
| 3 months | Clinical evaluation | Antibody titer, PCR test |
| 6 months | Infection and survival status | Antibody titer |
| 12 months | Final follow-up | Antibody titer, survival status |

COVID-19 infection is defined as PCR-confirmed SARS-CoV-2 positivity by nasopharyngeal swab or other approved method.

4.8 Data Collection and Quality Control

All data are recorded in case report forms (CRFs) and entered into a secure electronic database.

Double data entry and periodic quality audits are conducted by independent monitors.

Any protocol deviations or missing data are documented and reported.

**Ⅴ. Statistical Considerations**

5.1 Sample Size Determination

The sample size was calculated based on the expected difference in the incidence of COVID-19 infection between the CCP group and the control group.

According to preliminary clinical observations and published data, the infection rate within one year after HSCT was estimated at 80% in the control group and 50% in the CCP group.

Assuming a two-sided significance level (α = 0.05) and a statistical power of (1 – β = 0.80), and using a log-rank test for comparison between groups, the required total sample size was calculated to be 72 participants (36 per group).

This number also accounts for an estimated 10% attrition rate due to loss to follow-up or early withdrawal.

The sample-size calculation was performed using PASS software version 15.0 (NCSS, Kaysville, UT, USA).

5.2 Analysis Populations

Three analysis populations will be defined:

1. Intention-to-Treat (ITT) population:

Includes all randomized participants, analyzed according to the group to which they were originally assigned, regardless of treatment received.

2. Per-Protocol (PP) population:

Includes participants who complete all planned study procedures and at least four CCP infusions (for the CCP arm) without major protocol violations.

3. Safety population:

Includes all participants who receive at least one CCP infusion (in the experimental group) or standard care (in the control group), used for safety analyses.

5.3 Statistical Methods

1. Descriptive Statistics:

Continuous variables will be summarized as mean ± standard deviation (SD) or median (interquartile range, IQR) as appropriate.

Categorical variables will be summarized as counts and percentages.

2. Group Comparisons:

Continuous variables: Student’st-test (normally distributed) or Mann–WhitneyU test (non-normally distributed).

Categorical variables: Chi-square (χ²) or Fisher’s exact test.

Time-to-event data (e.g., infection-free survival): Kaplan–Meier analysis and log-rank test.

3. Multivariate Analysis:

Cox proportional-hazards regression will be used to identify independent factors associated with infection risk or survival.

Variables withP < 0.10 in univariate analysis will be included in the multivariate model.

4. Confidence Intervals:

Two-sided P values < 0.05 will be considered statistically significant.

For all main comparisons, 95% confidence intervals (CIs) will be reported alongside P values.

5. Correction for Multiple Comparisons:

When multiple secondary endpoints are analyzed,P values will be adjusted using the Benjamini–Hochberg false discovery rate (FDR) method to control for type I error.

5.4 Missing Data

No imputation will be performed for missing primary endpoint data.

Participants who withdraw before infection assessment will be censored at the date of last follow-up.

Sensitivity analyses will compare results between complete-case and ITT datasets to ensure robustness.

5.5 Interim and Safety Analysis

An interim safety review will be conducted after the first 20 participants complete all four CCP infusions.

The Data and Safety Monitoring Board (DSMB) will review adverse events, infection incidence, and survival data.

If any safety signal or unexpected event (e.g., transfusion-related acute lung injury) is detected, the DSMB may recommend protocol modification or early termination.

5.6 Softwarec

All statistical analyses will be performed using SPSS version 26.0 (IBM Corp., Armonk, NY, USA) and R software version 4.2.3 (R Foundation for Statistical Computing, Vienna, Austria).

Graphs and survival curves will be generated using GraphPad Prism 9.0 (GraphPad Software, San Diego, CA, USA).

**Ⅵ. Ethical Considerations**

6.1 Ethical Approval

This study was reviewed and approved by the Ethics Committee of Tianjin Institute of Hematology, Chinese Academy of Medical Sciences & Peking Union Medical College (Approval No. 2023-HSCT-CCP-06).

The protocol adheres to the principles outlined in the Declaration of Helsinki (2013 revision) and complies with the International Council for Harmonisation (ICH) Good Clinical Practice (E6 R2) and applicable Chinese national regulatory requirements for human-subject research.

No participant was enrolled prior to obtaining formal ethics approval.

6.2 Informed Consent

Before participation, each patient (or legally authorized representative) receives detailed verbal and written information regarding the study purpose, procedures, potential benefits, and foreseeable risks, including possible transfusion-related reactions.

Written informed consent must be obtained before any study-related procedure is performed.

Participants are informed of their right to withdraw from the study at any time without penalty or impact on their medical care.

All informed consent forms (ICFs) are stored in the investigator’s site file, and copies are provided to participants for their records.

6.3 Participant Safety and Risk Management

The primary potential risks involve transfusion-related reactions, including mild allergic reactions, febrile non-hemolytic transfusion reactions, or rare severe complications such as transfusion-related acute lung injury (TRALI).

All CCP infusions are conducted under physician supervision with continuous monitoring of vital signs.

If any Grade ≥3 adverse event occurs, the infusion is immediately discontinued, and appropriate medical management is initiated.

Any serious adverse event (SAE) suspected to be related to CCP infusion will be reported to the Ethics Committee and the Data and Safety Monitoring Board (DSMB) within 24 hours.

6.4 Confidentiality and Data Protection

All participant data are coded using unique study identification numbers.

Personal identifiers (e.g., name, ID number, contact information) are stored separately from study data in a secure, password-protected database accessible only to authorized personnel.

Any publication or presentation arising from this study will present aggregated data without revealing individual identities.

Data management complies with Chinese Personal Information Protection Law (2021) and institutional data protection policies.

6.5 Investigator Responsibilities

The Principal Investigator (PI) is responsible for:

1. Ensuring compliance with the approved protocol and ethical principles.
2. Overseeing data accuracy and integrity.
3. Reporting protocol deviations, SAEs, or amendments to the ethics committee.
4. Maintaining essential documents for at least five years after study completion or longer if required by regulation.

6.6 Publication and Data Sharing Policy

The study results, whether positive or negative, will be submitted for publication in peer-reviewed scientific journals.

Summary data may be shared with other researchers upon reasonable request, provided that confidentiality is maintained and data use complies with ethical and institutional guidelines.

6.7 Compensation and Insurance

All participants receive standard post-transplant medical care at no additional cost.

In the event of study-related injury or severe adverse event, treatment expenses are covered according to institutional policy and applicable national regulations.

**Ⅷ. References and Appendix Declaration**

8.1 Key References

1. World Health Organization. WHO Coronavirus (COVID-19) Dashboard. Updated December 2024.

2. Ljungman P, et al. Outcome of COVID-19 in hematopoietic stem-cell transplant recipients: An EBMT registry study. Transplant Cell Ther. 2021;27(9):772.e1–772.e7.

3. Murray SM, et al. Impaired humoral immunity to SARS-CoV-2 vaccination in hematopoietic stem cell transplant recipients. Br J Haematol. 2022;198(4):668–676.

4. Einarsdottir S, et al. Delayed lymphocyte reconstitution and hypogammaglobulinemia after allogeneic HSCT. Blood Adv. 2022;6(9):2723–2734.

5. Shanbhag S, et al. COVID-19 outcomes in hematopoietic cell transplant and CAR-T recipients: A CIBMTR report. Transplant Cell Ther. 2022;28(7):447.e1–447.e10.

6. Cao Y, et al. The Composite Immune Risk Score predicts overall survival after allogeneic hematopoietic stem-cell transplantation. Am J Hematol. 2023;98(2):309–319.

7. Vidarsson G, et al. Half-life and function of human IgG subclasses. Front Immunol. 2014;5:520.

8. EBMT Handbook, 10th Edition, 2024. Chapters “Supportive Care,” “Vaccination,” and “Follow-up after HCT.”

9. World Health Organization. Use of Convalescent Plasma in the Treatment of COVID-19: Interim Guidance. 2021.

10. National Health Commission of China. Good Clinical Practice for Investigational Drugs (GCP, 2020 Revision).

8.2 Abbreviations

| **Abbreviation** | **Full Term** |
| --- | --- |
| AE | Adverse Event |
| CCP | COVID-19 Convalescent Plasma |
| CI | Confidence Interval |
| CTCAE | Common Terminology Criteria for Adverse Events |
| DSMB | Data and Safety Monitoring Board |
| GVHD | Graft-versus-Host Disease |
| HSCT | Hematopoietic Stem Cell Transplantation |
| ICF | Informed Consent Form |
| ITT | Intention-to-Treat |
| PP | Per-Protocol |
| SAE | Serious Adverse Event |
| UDCA | Ursodeoxycholic Acid |
| WHO | World Health Organization |

8.3 Document Approval and Version Control

| **Item** | **Description** |
| --- | --- |
| Protocol Title | *Prophylactic Use of COVID-19 Convalescent Plasma in Adult Patients Early After Hematopoietic Stem Cell Transplantation (CCP-COVID-19-HSCT-2023)* |
| Version | 6.0 |
| Date | January 7, 2023 |
| Principal Investigator | **Dr. Yigeng Cao** |
| Institution | Department of Hematopoietic Stem Cell Transplantation, Tianjin Institute of Hematology, Chinese Academy of Medical Sciences & Peking Union Medical College |
| Ethics Approval No. | 2023-HSCT-CCP-06 |

8.4 Appendix Declaration

This English document is an official translation of the original Chinese protocol titled “CCP-COVID-19-HSCT-2023-V6.0,” approved on January 7, 2023, by the Ethics Committee of Tianjin Institute of Hematology, CAMS & PUMC. The original Chinese version is the authoritative text. Copies of the original document are available upon request.
